# Progress analysis of a multi-recombinative evolution strategy on the highly multimodal Rastrigin function✩

**DOI:** 10.1016/j.tcs.2023.114179

**Published:** 2023-09-22

**Authors:** Amir Omeradzic, Hans-Georg Beyer

**Affiliations:** Vorarlberg University of Applied Sciences, Research Center Business Informatics, Hochschulstraße 1, 6850 Dornbirn, Austria

**Keywords:** Evolution strategy, Progress rate, Global optimization, Rastrigin function

## Abstract

A first and second order progress rate analysis was conducted for the intermediate multi-recombinative Evolution Strategy (*μ*/*μ_I_*, λ)-ES with isotropic scale-invariant mutations on the highly multimodal Rastrigin test function. Closed-form analytic solutions for the progress rates are obtained in the limit of large dimensionality and large populations. The first order results are able to model the one-generation progress including local attraction phenomena. Furthermore, a second order progress rate is derived yielding additional correction terms and further improving the progress model. The obtained results are compared to simulations and show good agreement, even for moderately large populations and dimensionality. The progress rates are applied within a dynamical systems approach, which models the evolution using difference equations. The obtained dynamics are compared to real averaged optimization runs and yield good agreement. The results improve further when dimensionality and population size are increased. Local and global convergence is investigated within given model showing that large mutations are needed to maximize the probability of global convergence, which comes at the expense of efficiency. An outlook regarding future research goals is provided.

## 1 Introduction

The theoretical analysis of the performance of Evolution Strategies (ES) [8] optimizing functions *f* (**y**) in real-valued *N*-dimensional search spaces **y** ∈ ℝ^*N*^ is a challenge. This is due to the probabilistic nature of these algorithms allowing up to now the dynamic progress analysis only on simple test functions such as the sphere model [2,5], the ridge function class [3,14], and the ellipsoid model [7]. These test functions are simple w.r.t. their optimization landscape (also referred to as fitness landscape) in that they have at most one optimizer (i.e., the location **y** of the optimum). Analyzing the dynamical behavior of ES on more complex and multimodal test functions appears to be even more demanding. However, ES and other evolutionary algorithms are especially designated to optimize such problems. There is empirical evidence that ES are able to globally optimize highly multimodal optimization problems [11] with in *N* exponential number of local optima. The question arises how and when these ES are able to locate the global optimizer. It is the long term goal to find conditions the ES must fulfill to not get trapped in the vast amount of local optimizers. Ideally, a theoretical analysis should provide the answers regarding the success probability *P_S_* (of locating the global optimum) depending on the ES parameters such as the population size λ and the test function to be optimized. Furthermore, one is interested in the computational complexity of the optimization process.

One approach successfully applied to the analysis of the ES-performance on simple unimodal test functions mentioned above is the dynamical systems approach [5] which is based on progress rate analysis. The progress rate is a measure of expected positional change in search space between two generations depending on location, strategy and test function parameters. The idea of investigating global search behavior from expected local progress was successfully applied, among others, in [3,7]. It will be shown in this paper that this approach can be extended to the highly multimodal Rastrigin test function

(1) $$f(\text{y})=\sum_{i=1}^{N}f_{i}\left(y_{i}\right)=\sum_{i=1}^{N}\left[y_{i}^{2}+A(1-\cos(\alpha \;y_{i}))\right],$$

where **y** ∈ ℝ^*N*^, with oscillation amplitude *A* and frequency parameter *α*. The *i*-th fitness component in Eq. (1) is defined as

(2) $$f_{i}(y_{i}):=y_{i}^{2}+A(1-\cos(\alpha \;y_{i})).$$

**Fig. 1:**
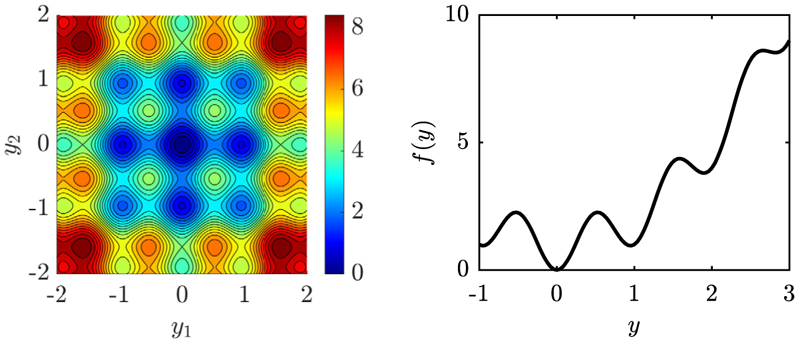
The heat map shows the optimization landscape for *A* = 1, *α* = 2*π*, and *N* = 2. The global minimizer located at the origin (dark blue) is surrounded by multiple local minima. On the right side the same parameter set is shown for *N* = 1. For increasing *y* the oscillation contribution is decreasing. (For interpretation of the colors in the figure(s), the reader is referred to the web version of this article.)

Depending on *A* and *α* a finite number of local minima *M* can be observed for each component *i*. Therefore, the overall number of local minima is scaling as *M^N^* posing a highly multimodal minimization problem with the global optimizer located at **ŷ** = **0**. An exemplary optimization landscape of the Rastrigin function is shown in Fig. 1.

The remarkable observation is that ES – unlike classical nonlinear optimization algorithms (e.g. BFGS) – do not follow the local gradient or Hessian ending in one of the *M^N^* − 1 local optimizers. That is, ES perform a rather global search. A deeper understanding of this behavior is still missing. Recently, attempts have been made to analyze the problem from the viewpoint of relaxation using kernel smoothing [15]. However, the sampling process needed to transform the original problem into a convex optimization problem is still lacking a link to the ES.

In this paper a simplified and scale-invariant (*μ*/*μ_I_*, λ)-ES, see Algorithm 1, is analyzed with step-size control defined in Eq. (4). Starting from the so-called parental centroid vector **y**^(*g*)^ a population of λ offspring are generated by adding isotropic Gaussian mutations **x** ~ *σ*𝒩(**0**, **1**) with mutation strength *σ* in Lines 6 and 7. Thereafter, the fitness is evaluated in Line 8. Selection of the *μ* best individuals is done in Line 10. It is performed for a given selection (truncation) ratio defined as

(3) $$\vartheta :=\frac{\mu }{\lambda },$$

with *ϑ* ∈ (0, 1). It will be an essential quantity for the progress rate results in the limit of large population sizes. Using intermediate recombination with equal weights the best *m* = 1, …, *μ* individuals are recombined in Line 11 and the new parental centroid **y**^(*g*+1)^ is obtained. In the following, the subscript “*m*; λ” can be read as the *m*-th best solution out of λ candidate solutions. In Line 12 the simplified step-size adaptation is performed. To this end, a constant normalized mutation σ* using the spherical normalization with ‖**y**^(*g*)^‖ = *R*^(*g*)^ is defined as

(4) $$\sigma^{*}:=\frac{\sigma^{\left(g\right)}N}{\left\|{\text{y}}^{\left(g\right)}\right\|}=\frac{\sigma^{\left(g\right)}N}{R^{\left(g\right)}}.$$

This property ensures scale invariance and therefore global convergence of the algorithm, as the mutation strength *σ*^(*g*)^ decreases if and only if the residual distance *R*^(*g*)^ decreases. The quantity σ* is unknown during black-box optimizations, but it is very useful for theoretical investigations to obtain scale-invariant mutations strengths.

Algorithm 1 (*μ/μ_I_*, λ)-ES with constant σ*.

1: *g* ← 0

2: **y**^(0)^ ← **y**^(init)^

3: *σ*^(0)^ ← σ*‖**y**^(0)^‖/*N*

4: **repeat**

5:    **for** *l* ← 1, …, λ **do**

6:            ${\tilde{x}}_{l}\leftarrow \sigma^{\left(g\right)}{𝒩}_{l}(0,1)$

7:            ${\tilde{y}}_{l}\leftarrow {\text{y}}^{\left(g\right)}+{\tilde{x}}_{l}$

8:            ${\tilde{f}}_{l}\leftarrow f\left({\tilde{y}}_{l}\right)$

9:    **end for**

10:    (**ỹ**_1;λ_,…, **ỹ**_*μ*;λ_) ← sort (**ỹ** w.r.t. ascending $\tilde{f}$)

11:    ${\text{y}}^{\left(\text{g}+1\right)}\leftarrow \frac{1}{\mu }\sum_{m=1}^{\mu }{\tilde{y}}_{m;\lambda }$

12:    *σ*^(*g*+1)^ ← σ*‖**y**^(*g*+1)^‖/*N*

13:    *g* ← *g* + 1

14: **until** termination criterion

The remainder of this paper is organized as follows. In the next section the local performance measures will be introduced being the basis for both the progress rate analysis and the dynamical systems approach. Section 3 is devoted to the determination and evaluation of the first order progress rate. Section 4 describes the derivation of the second order progress rate, which will rely on first order progress rate results. Section 5 uses the local performance measures to establish the evolution equations that govern the dynamical behavior of the ES. Experiments will be presented to show the usefulness of the approach. In the final Section 6 conclusions will be drawn and being based on open problems the further research direction will be outlined.

## 2 Local performance measures and quality gain distribution

The performance of an ES between two generations can be evaluated in both fitness and search space. The quality gain *Q*_**y**_(**x**) of fitness *f* at a position **y**^(*g*)^ due to an isotropic mutation **x** ~ *σ*𝒩(**0**, **1**) is defined as

(5) $$Q_{\text{y}}(\text{x}):=f\left({\text{y}}^{\left(g\right)}+\text{x}\right)-f\left({\text{y}}^{\left(g\right)}\right),$$

and yields in the case of fitness improvement (minimization considered) a negative value *Q*_**y**_ < 0. The definition (5) measures the fitness change before selection and will be needed for the evaluation of the two progress rates (7) and (8). The quality gain components are decomposed using *f_i_* from Eq. (2) as *Q_i_* := *f_i_*(*y_i_* + *x_i_*) − *f_i_*(*y_i_*), such that

(6) $$Q_{\text{y}}(\text{x})=\sum_{i=1}^{N}Q_{i}(x_{i})=\sum_{i=1}^{N}\left[f_{i}\left(y_{i}^{\left(g\right)}+x_{i}\right)-f_{i}\left(y_{i}^{\left(g\right)}\right)\right].$$

That is, the quality gain corresponds to the difference between fitness values before and after the mutation application. A probabilistic model for the distribution of quality values will be presented below. It will be important for the subsequent progress rate derivations, as selection is based on fitness values.

Analyzing the progress towards the optimizer in search space, the first order progress rate on the Rastrigin function has already been investigated in [17] as a first approach. In this paper, a new approach is presented which significantly improves the prediction quality.

The first order progress rate between two generations for the parental component *y_i_* is defined as

(7) $$\varphi_{i}:=\text{E}\left[y_{i}^{\left(g\right)}-y_{i}^{\left(g+1\right)}|{\text{y}}^{\left(g\right)},\sigma^{\left(g\right)}\right],$$

given parental position **y**^(*g*)^ and mutation strength *σ*^(*g*)^ at generation *g*. It is a measure of expected positional difference in search space. Positive expected progress *φ_i_* > 0 is defined in the case $y_{i}^{\left(g\right)}>\text{E}\left[y_{i}^{\left(g+1\right)}\right]$ for $y_{i}^{\left(g\right)}>0$ and $\text{E}\left[y_{i}^{\left(g+1\right)}\right]>0$. In this case the distance to the optimizer *ŷ_i_* = 0 is reduced in expectation. This assumption is only valid as long as the sign of $\text{E}\left[y_{i}^{\left(g+1\right)}\right]$ does not change, i.e., for small mutations compared to the residual distance. Therefore *φ_i_* has limited applicability when studying the convergence behavior in the vicinity of the optimizer. As has been shown in [7] regarding the performance analysis on the ellipsoid model, a second order progress rate is needed. It is defined as

(8) $$\varphi_{i}^{\text{II}}:=\text{E}\left[{\left(y_{i}^{\left(g\right)}\right)}^{2}-{\left(y_{i}^{\left(g+1\right)}\right)}^{2}|{\text{y}}^{\left(g\right)},\sigma^{\left(g\right)}\right].$$

Squaring the positions yields $\varphi_{i}^{\text{II}}>0$ independent of the sign, if the distance to *ŷ_i_* = 0 decreases in expectation. Additionally, the derivation will yield expressions containing a progress gain and loss part, which is necessary for a more accurate model of convergence. Both progress rates will be expressed using integral equations for the expected values and approximations will be necessary to find closed-form solutions. In a second step the progress rates can be applied within difference equations to model the expected dynamics over many generations in order to investigate the global convergence behavior.

**Fig. 2:**
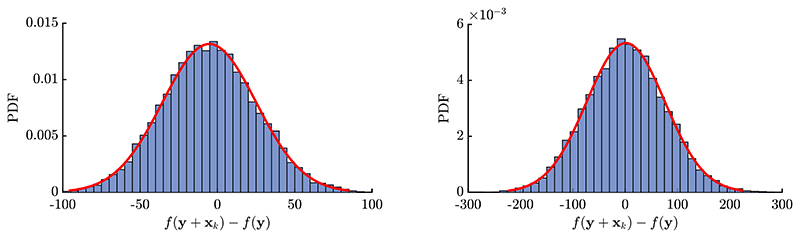
The histograms show sampled values of *Q*_**y**_(**x**) from (5) with fixed **y** by applying random mutations **x**_*k*_ ~ *σ*𝒩(**0**, **1**) (*σ* = 1 with *k* = 1, …, 10^4^ samples) at *N* = 10 (left) and *N* = 100 (right) with *A* = 10. The **y**-values were initialized randomly at ‖**y**‖ = 10 where local attraction is significant. The red envelope curves show the respective normal approximation (9) using mean value (30) and variance (31). The *p*-values of the Anderson-Darling-test for normality are *p* = 0.48 (left) and *p* = 0.53 (right).

The selection of individuals is based on the attained fitness values. The quality gain measures the fitness change before selection according to (5). When the progress rate of an ES is modeled, the cumulative distribution function (CDF) *P_Q_* (*q*) of the quality gain and its probability density function (PDF) *p_Q_* (*q*) are needed as a function of **y** and *σ*. Obtaining an exact CDF for *Q*_**y**_(**x**) is not feasible at this point. Since $Q_{\text{y}}(\text{x})=\sum_{i=1}^{N}Q_{i}\left(x_{i}\right)$ with independent random variables *Q_i_*, the application of the Central Limit Theorem seems appropriate to show that the distribution is asymptotically normal.^1^ However, proving its validity rigorously seems hard or even impossible for arbitrary **y**. Therefore, we resort to normality as an approximation for the quality gain distribution. This is backed up by experimental results in Fig. 2, where sampled *Q*_**y**_(**x**)-values are compared to the normal approximation. A standard Anderson-Darling test was performed to check whether the sampled data was drawn from a normal distribution with known mean and variance according to (9). The hypothesis test fails to reject the normality assumption at *p*-values *p* = 0.48 (left) and *p* = 0.53 (right), where rejection is usually defined for *p* < 0.05. Even at relatively small *N* = 10 the results agree well. Good experimental agreement is also observed for the variation of the location **y** and mutation strength *σ* (not shown). Therefore, the normality assumption does not pose a strong restriction on the overall prediction quality of the progress rates in the subsequent sections, such that we approximate

(9) $$Q_{\text{y}}(\text{x})=\sum_{i=1}^{N}Q_{i}(x_{i})\sim 𝒩(\text{E}\left[Q_{\text{y}}(\text{x})\right],\text{Var}\left[Q_{\text{y}}(\text{x})\right]).$$

Furthermore, the following abbreviations are introduced

(10) $$E_{Q}:=\text{E}\left[Q_{\text{y}}(\text{x})\right]=\sum_{i=1}^{N}\text{E}\left[Q_{i}\right]$$

(11) $$D_{Q}^{2}:=\text{Var}\left[Q_{\text{y}}(\text{x})\right]=\sum_{i=1}^{N}\text{Var}\left[Q_{i}\right].$$

At this point an additional assumption for the coordinates **y** = (*y*_1_, …, *y_N_*) has to be made to justify subsequent variance approximations (13) and (14). Given the search vector **y** = (*y*_1_, …, *y_N_*) and residual distance *R*^2^ = ‖**y**‖^2^ it is assumed that the components contribute *approximately* equally (in expectation) to the residual distance, i.e., there is no dominating component, such that

(12) $$\frac{y_{i}^{2}}{R^{2}}\approx \frac{1}{N},\;\text{for}\;\text{all}\;i=1,\ldots ,N.$$

Property (12) will also be referred to as *component equipartition*. The concept was introduced in [6] and proven for the noisy ellipsoid in [12]. Its applicability to the Rastrigin function was shown in [19]. The equipartition assumption is necessary in order to justify certain approximation steps and to provide a closed-form solution for the progress rate. Furthermore, it will be a reasonable assumption to obtain a model of the algorithm’s progress and dynamics in expectation. This assumption also justifies a linear scaling of the variance with dimensionality *N* provided that the components are contributing equally to the overall variance, such that

(13) $$D_{Q}^{2}=\sum_{i=1}^{N}\text{Var}\left[Q_{i}\right]=\Theta (N).$$

Additionally, for large *N* an important approximation will be used for the variance to significantly simplify the obtained lengthy results. If no single *i*-th component is dominating the sum, i.e., Var [*Q_i_*] / Σ_*j*≠*i*_ Var [*Q_j_*] → 0 (for any *i* in the limit *N* → ∞), the contribution of a single term is negligible for *N* → ∞. Therefore, the two sums over *N* and *N* − 1 terms, respectively, are asymptotically equal with

(14) $$D_{Q}^{2}=\sum_{i=1}^{N}\text{Var}\left[Q_{i}\right]≃\sum_{j\neq i}\text{Var}\left[Q_{j}\right]=D_{i}^{2}.$$

Note that quantity $D_{i}^{2}$ is formally introduced in (20). Returning to Eq. (9), the expression is rewritten using a standardized random variate *Z* as

(15) $$Z=\frac{Q_{\text{y}}(\text{x})-E_{Q}}{D_{Q}}\overset{N\to \infty }{\sim }𝒩(0,1).$$

### Approximation 1 *(Quality gain distribution)*

The local quality gain at position **y** due to random mutation vector **x** ~ 𝒩(**0**, *σ*^2^**1**) is approximately normally distributed. Therefore, *P_Q_* (*q*) and *p_Q_* (*q*) can be approximated as

(16) $${\tilde{P}}_{Q}\;(q)=\Phi \left(\frac{q-E_{Q}}{D_{Q}}\right)$$

(17) $${\tilde{p}}_{Q}\;(q)=\frac{1}{\sqrt{2\pi }\;D_{Q}}\exp\left[-\frac{1}{2}{\left(\frac{q-E_{Q}}{D_{Q}}\right)}^{2}\right].$$

Within the normal approximation (16) the inverse ${\tilde{P}}_{Q}^{-1}(p)$ given some probability *p* can be easily obtained by using the quantile function Φ^−1^(*p*) of the normal distribution. This relation will be used later to obtain a quality gain for some given probability *p* using

(18) $$q=E_{Q}+D_{Q}\Phi^{-1}(p).$$

For the derivation of the *i*-th component progress rate the conditional distribution function *P_Q_* (*q*|*x_i_*) of the quality gain is needed for a given component *x_i_*. In this case expected value and variance are given by

(19) $$E_{Q|x_{i}}:=\text{E}\left[Q_{\text{y}}(\text{x})|x_{i}\right]=Q_{i}\left(x_{i}\right)+\sum_{j\neq i}\text{E}\left[Q_{j}\right]$$

(20) $$D_{i}^{2}:=\text{Var}\left[Q_{\text{y}}(\text{x})|x_{i}\right]=\sum_{j\neq i}\text{Var}[Q_{j}],$$

where the sum *j* ≠ *i* is taken for fixed *i* over the remaining *N* − 1 components. Therefore, a normal approximation for the conditional CDF is introduced using (19) and (20).

### Approximation 2 (*Quality gain distribution given x_i_*)

The quality gain distribution at position **y** given fixed mutation component *x_i_* and random mutation vector (**x**)_*j*≠*i*_ ~ (𝒩(**0**, *σ*^2^**1**))_*j*≠*i*_ is approximately normally distributed. Therefore, *P_Q_* (*q*|*x_i_*) and *p_Q_* (*q*|*x_i_*) can be approximated as

(21) $${\tilde{P}}_{Q}\left(q|x_{i}\right)=\Phi \left(\frac{q-E_{Q|x_{i}}}{D_{i}}\right)$$

(22) $${\tilde{P}}_{Q}\left(q|x_{i}\right)=\frac{1}{\sqrt{2\pi }D_{i}}\exp\left[-\frac{1}{2}{\left(\frac{q-E_{Q|x_{i}}}{D_{i}}\right)}^{2}\right].$$

Having derived approximations of the quality gain distribution functions, the quantities E [*Q_i_*] and Var [*Q_i_*] remain to be determined. As the components are independent, it is sufficient to consider a single component and then perform the summation. Starting from definition (6), one can evaluate the quality gain of a single component *Q_i_*(*x_i_*). After applying trigonometric identity cos (*α*(*y_i_* + *x_i_*)) = cos (*αy_i_*) cos (*αx_i_*) − sin (*αy_i_*) sin (*αx_i_*), one gets

(23) $$Q_{i}(x_{i})=f_{i}(y_{i}+x_{i})-f_{i}(y_{i})$$

(24) $$=x_{i}^{2}+2y_{i}x_{i}+A\cos(\alpha y_{i})-A\cos(\alpha y_{i})\cos(\alpha x_{i})+A\sin(\alpha y_{i})\sin(\alpha x_{i})\text{,}$$

of which E [*Q_i_*] and $\text{Var}\left[Q_{i}\right]=\text{E}\left[Q_{i}^{2}\right]-\text{E}{\left[Q_{i}\right]}^{2}$ need to be evaluated. The results will be expressed as expected values containing trigonometric functions. As a remark, terms containing moments of *x_i_* ~ 𝒩(0, *σ*^2^), i.e., $\text{E}\left[x_{i}^{k}\right]$ with *k* ≥ 1, are silently evaluated as they are assumed to be widely known. Starting with E[*Q_i_*] one has

(25) $$\text{E[}Q_{i}\text{]}=\sigma^{2}+A\cos(\alpha y_{i})(1-\text{E[}\cos(\alpha x_{i})\text{]}),$$

where odd powers of $\text{E}\left[x_{i}^{k}\right]=0$, which also yields E[sin (*αx_i_*)] = 0. Evaluating Var [*Q_i_*] yields

(26) $$\begin{array}{l} \text{Var}[Q_{i}]=\text{E}\left[Q_{i}^{2}\right]-\text{E[}Q_{i}{\text{]}}^{2}\\ \;=2\sigma^{4}+4y_{i}^{2}\sigma^{2}+A^{2}\sin^{2}(\alpha y_{i})\text{Var}[\sin(\alpha x_{i})]\\ \;+A^{2}\cos^{2}(\alpha y_{i})\text{Var}[\cos(\alpha x_{i})]-2A\cos(\alpha y_{i})\text{E}\left[x_{i}^{2}\cos(\alpha x_{i})\right]\\ \;+2A\sigma^{2}\cos(\alpha y_{i})\text{E[}\cos(\alpha x_{i})\text{]}+4Ay_{i}\sin(\alpha y_{i})\text{E[}x_{i}\sin(\alpha x_{i})\text{]}. \end{array}$$

Expectations of the form $\text{E}\left[x_{i}^{k}\cos\alpha x_{i}\right]$ and $\text{E}\left[x_{i}^{k}\sin\alpha x_{i}\right]$ for *k* ≥ 0 can be obtained by using the definition of the characteristic function *χ* of a random variate *x* ~ 𝒩(*μ*, *σ*^2^) and its known result [1]

(27) $$\chi_{x}(\alpha )=\text{E}\left[{\text{e}}^{ı\alpha x}\right]={\text{e}}^{ı\alpha \mu -\frac{1}{2}\alpha^{2}\sigma^{2}}={\text{e}}^{-\frac{1}{2}\alpha^{2}\sigma^{2}}[\cos(\alpha \mu )+ı\sin(\alpha \mu )],$$

with the imaginary unit denoted by $ı=\sqrt{-1}$ in (27) and (28). Now the *k*-th derivatives with respect to *α* can be applied to both sides

(28) $$\begin{array}{l} \frac{{\text{d}}^{k}}{\;\text{d}\alpha^{k}}\text{E}\left[{\text{e}}^{ı\alpha x}\right]=\text{E}\left[\frac{{\text{d}}^{k}}{\;\text{d}\alpha^{k}}{\text{e}}^{ı\alpha x}\right]=\text{E}\left[\frac{{\text{d}}^{k}}{\;\text{d}\alpha^{k}}\cos(\alpha x)\right]+ı\text{E}\left[\frac{{\text{d}}^{k}}{\;\text{d}\alpha^{k}}\sin(\alpha x)\right]\\ \;\overset{!}{=}\frac{{\text{d}}^{k}}{\;\text{d}\alpha^{k}}\left[{\text{e}}^{-\frac{{\left(\alpha \sigma \right)}^{2}}{2}}[\cos(\alpha \mu )+ı\sin(\alpha \mu )]\right], \end{array}$$

such that corresponding real and imaginary parts can be identified by comparing both sides (denoted by $\underset{=}{!}$) of Eq. (28). Given *μ* = 0 for *k* = {0, 1, 2} the required expectations of trigonometric terms can be derived. Additionally, trigonometric identities cos^2^(*x*) = 1/2 + cos(2*x*)/2 and sin^2^(*x*) = 1/2 − cos(2*x*)/2 are used. The results are

(29) $$\begin{array}{c} \;\text{E}[\cos(\alpha x)]={\text{e}}^{-\frac{{\left(\alpha \sigma \right)}^{2}}{2}},\;\text{E}\left[\cos^{2}(\alpha x)\right]=\frac{1}{2}+\frac{1}{2}{\text{e}}^{-\frac{{\left(2\alpha \sigma \right)}^{2}}{2}}\\ \;\text{E}\left[\sin^{2}(\alpha x)\right]=\frac{1}{2}-\frac{1}{2}{\text{e}}^{-\frac{{\left(2\alpha \sigma \right)}^{2}}{2}},\;\text{E}[x\sin(\alpha x)]=\alpha \sigma^{2}{\text{e}}^{-\frac{{\left(\alpha \sigma \right)}^{2}}{2}}\\ \text{E}\left[x^{2}\cos(\alpha x)\right]=(\sigma^{2}-\alpha^{2}\sigma^{4}){\text{e}}^{-\frac{{\left(\alpha \sigma \right)}^{2}}{2}},\;\text{Var}[(\cdot )]=\text{E}\left[{\left(\cdot \right)}^{2}\right]-\text{E}{\left[(\cdot )\right]}^{2}. \end{array}$$

Inserting relations (29) into (25) and (26), summing over all *N* components and collecting the resulting terms one obtains the expected value

(30) $$E_{Q}=\sum_{i=1}^{N}\left[\sigma^{2}+A\cos(\alpha y_{i})\left(1-{\text{e}}^{-\frac{{\left(\alpha \sigma \right)}^{2}}{2}}\right)\right].$$

Analogously, the variance of the Rastrigin quality gain yields

(31) $$\begin{array}{lll} D_{Q}^{2} & = & \sum_{i=1}^{N}[4\sigma^{2}y_{i}^{2}+2\sigma^{4}+\frac{A^{2}}{2}\left(1-{\text{e}}^{-{\left(\alpha \sigma \right)}^{2}}\right)\left(1-\cos(2\alpha y_{i}){\text{e}}^{-{\left(\alpha \sigma \right)}^{2}}\right)\\ & & +2A\alpha \sigma^{2}{\text{e}}^{-\frac{1}{2}{\left(\alpha \sigma \right)}^{2}}\left(\alpha \sigma^{2}\cos(\alpha y_{i})+2y_{i}\sin(\alpha y_{i})\right)]. \end{array}$$

The quantities *E_Q|x_i__* from (19) and $D_{i}^{2}$ from (20) are given analogously by summing over *N* − 1 components. Expressions *E_Q_* and *D_Q_* could be inserted into (16), and *E_Q|x_i__* with *Q_i_*(*x_i_*) and *D_i_* into (21). However, it is omitted at this point for better readability.

As an important remark, expression (23) can be linearized w.r.t. mutation *x_i_* to obtain analytically solvable progress rate integrals, see also discussion after Eq. (51). Taylor-expanding *f_i_* around *y_i_* for small *x_i_* gives $f_{i}\left(y_{i}+x_{i}\right)=f_{i}\left(y_{i}\right)+\frac{\partial f_{i}}{\partial y_{i}}x_{i}+O\left(x_{i}^{2}\right)$, such that after setting $f_{i}^{\prime }:=\frac{\partial f_{i}}{\partial y_{i}}$ and evaluating the derivative one has

(32) $$\begin{array}{l} Q_{i}(x_{i})=f_{i}(y_{i}+x_{i})-f_{i}(y_{i})=f_{i}^{\prime }x_{i}+O\left(x_{i}^{2}\right)\\ \;=(2y_{i}+\alpha A\sin(\alpha y_{i}))x_{i}+O\left(x_{i}^{2}\right)=(k_{i}+d_{i})x_{i}+O\left(x_{i}^{2}\right), \end{array}$$

with following definitions applied to (32)

(33) $$f_{i}^{\prime }:=k_{i}+d_{i},\;\text{with}\;k_{i}:=2y_{i},\;\text{and}\;d_{i}:=\alpha A\sin(\alpha y_{i}).$$

Component *k_i_* is the derivative of the quadratic term $y_{i}^{2}$, cf. Eq. (2), which follows the global quadratic structure of the function. Conversely, derivative *d_i_* follows the local oscillation, such that it will be very important for the model of local attraction during the progress rate derivations in Secs. 3 and 4.

## 3 First order progress rate

While the first order progress rate (7) does not suffice to completely describe the convergence behavior of the ES on Rastrigin, see Sec. 5, it is a necessary step in the calculation of the second order progress rate in Sec. 4. Given definition (7) and the parental location **y**^(*g*)^, one has to find the expected value over the *i*-component location $\text{E}\left[y_{i}^{\left(g+1\right)}\right]$. The positional update **y**^(*g*)^ → **y**^(*g*+1)^ performed by the ES is realized by consecutively applying mutation, selection, and recombination (see Algorithm 1), such that one can write

(34) $${\text{y}}^{\left(g+1\right)}=\frac{1}{\mu }\sum_{m=1}^{\mu }\left({\text{y}}^{\left(g\right)}+{\text{x}}_{m;\text{λ}}\right)={\text{y}}^{\left(g\right)}+\frac{1}{\mu }\sum_{m=1}^{\mu }{\text{x}}_{m;\text{λ}},$$

where **x**_*m*;λ_ denotes the mutation vector of the *m*-th best offspring after selection. Considering the *i*-th component of Eq. (34), abbreviating the mutation component as *x*_*m*;λ_ := (**x**_*m*;λ_)*_i_*, and taking the expected value thereof yields

(35) $$\text{E}\left[y_{i}^{\left(g+1\right)}|{\text{y}}^{\left(g\right)},\sigma^{\left(g\right)}\right]=y_{i}^{\left(g\right)}+\frac{1}{\mu }\sum_{m=1}^{\mu }\text{E}\left[x_{m;\text{λ}}|{\text{y}}^{\left(g\right)},\sigma^{\left(g\right)}\right].$$

The progress rate can therefore be evaluated by inserting (35) into (7) giving

(36) $$\varphi_{i}=-\frac{1}{\mu }\sum_{m=1}^{\mu }\text{E}\left[x_{m;\text{λ}}|{\text{y}}^{\left(g\right)},\sigma^{\left(g\right)}\right].$$

Before starting the derivation of (36), the important large population theorem is stated which will be used during the derivation of both first and second order progress rate. Its application also yields the so-called asymptotic generalized progress coefficients presented in Eq. (45).

### Theorem 1

*Let* λ *> μ* + 1 *and μ > a with a* ≥ 1 *and ϑ = μ/*λ *with* 0 < *ϑ* < 1, *such that t*^λ−*μ*−1^(1 − *t*)*^μ−a^ exhibits its maximum on* (0, 1) *and vanishes at t* ∈ {0, 1}. *Let f_x_(t) be a function defined for constant x* ∈ ℝ, *such that f_x_* : [0, 1] → [0, 1] *with bounded derivatives on* [0, 1] *and let* B *denote the beta function. Furthermore, let p_x_ denote the PDF of a normally distributed variate and let p_n_(x) denote a polynomial of degree n in x. For infinitely large μ,* λ → ∞ *and constant ϑ = μ*/λ *the following limit holds*

(37) $$\begin{array}{ll} \underset{\begin{array}{c} \mu ,\lambda \to \infty \\ \vartheta =\text{const}. \end{array}}{\lim} & \int_{-\infty }^{\infty }p_{n}(x)p_{x}(x)\frac{1}{\;\text{B}(\lambda -\mu ,\mu )}\int_{0}^{1}t^{\lambda -\mu -1}{\left(1-t\right)}^{\mu -a}f_{x}(t)\text{d}t\;\text{d}x\\ & =\frac{1}{\vartheta^{a-1}}\int_{-\infty }^{\infty }p_{n}(x)p_{x}(x)f_{x}(1-\vartheta )\text{d}x. \end{array}$$

### Proof

The dominated convergence theorem is applied. First, the following sequence is defined for *μ* = 1, 2, …, with λ(*μ*) = *μ*/*ϑ* and constant *ϑ*

(38) $$g_{\mu }(x):=\frac{1}{\;\text{B}(\text{λ}-\mu ,\mu )}\int_{0}^{1}t^{\text{λ}-\mu -1}(1-t{)}^{\mu -a}f_{x}(t)\text{d}t.$$

Note that *g_μ_* is measured over the density of the normal distribution. In [18] it was shown that *g_μ_*(*x*) converges for any *x* according to

(39) $$\underset{\begin{array}{c} \mu ,\text{λ}\to \infty \\ \vartheta =\text{ const}. \end{array}}{\lim}g_{\mu }(x)=\frac{f_{x}(1-\vartheta )}{\vartheta^{a-1}}.$$

An upper bound of *g_μ_* can be estimated using 0 ≤ *f_x_* ≤ 1 and the definition of the beta function $\text{B}(z_{1},\;z_{2})=\int_{0}^{1}t^{z_{1}-1}{\left(1-t\right)}^{z_{2}-1}\text{d}t$ as

(40) $$\begin{array}{l} \left|g_{\mu }(x)\right|\leq \frac{\text{B}(\text{λ}-\mu ,\mu -a+1)}{\text{B}(\text{λ}-\mu ,\mu )}=\frac{(\text{λ}-\mu -1)!(\mu -a)!}{(\text{λ}-a)!}\frac{(\text{λ}-1)!}{(\text{λ}-\mu -1)!(\mu -1)!}\\ \;=\frac{(\text{λ}-1)(\text{λ}-2)\cdots (\text{λ}-a+1)(\text{λ}-a)!}{(\mu -1)(\mu -2)\cdots (\mu -a+1)(\mu -a)!}\frac{(\mu -a)!}{(\text{λ}-a)!}\\ \;={\left(\frac{\text{λ}}{\mu }\right)}^{a-1}\frac{\left(1-1/\text{λ})\cdots (1-(a-1)/\text{λ}\right)}{\left(1-1/\mu )\cdots (1-(a-1)/\mu \right)}\\ \;\leq \frac{1}{\vartheta^{a-1}}\frac{1}{{\left(1-(a-1)/\mu \right)}^{a-1}}. \end{array}$$

A lower bound for the denominator of (40) can be given as

(41) $${\left(1-\frac{a-1}{\mu }\right)}^{a-1}\geq \frac{1}{a^{a-1}}.$$

Inequality (41) can be shown easily by setting *μ* = *a* + *k* with integers *a* ≥ 1 and *k* ≥ 1 (ensuring *μ* > *a*). This yields

(42) $$\begin{array}{r} 1-\frac{a-1}{\mu }=\frac{a+k-a+1}{a+k}\geq \frac{1}{a}\\ ak\geq k\text{, } \end{array}$$

which is fulfilled for any *a* ≥ 1 and *k* ≥ 1. Using (41) in (40) one gets

(43) $$\left|g_{\mu }(x)\right|\leq {\left(\frac{a}{\vartheta }\right)}^{a-1}.$$

As there is a constant upper bound of |*g_μ_*(*x*)|, it remains to show that

(44) $$\begin{array}{l} \int_{-\infty }^{\infty }\left|p_{n}(x)\right|p_{x}(x)\text{d}x\leq \int_{-\infty }^{\infty }\left|\sum_{k=0}^{n}a_{k}x^{k}\right|p_{x}(x)\text{d}x\leq \int_{-\infty }^{\infty }\sum_{k=0}^{n}\left|a_{k}\right|\left|x^{k}\right|p_{x}(x)\text{d}x\\ \;\leq 2\sum_{k=0}^{n}\left|a_{k}\right|\int_{0}^{\infty }x^{k}p_{x}(x)\text{d}x<\infty , \end{array}$$

which is finite due to normal density *p_x_*(*x*). Hence, the limit in Eq. (37) can be exchanged with the integral over *x*. Using the limit of (39) the desired result is obtained.

The limit (39) is readily used in [16] to define the so-called asymptotic generalized progress coefficients for integers *a* ≥ 1, *b* ≥ 0, and truncation ratio 0 < *ϑ* < 1 as

(45) $$e_{\vartheta }^{a,b}:={\left[\frac{{\text{e}}^{-\frac{1}{2}{\left[\Phi^{-1}(\vartheta )\right]}^{2}}}{\sqrt{2\pi }\vartheta }\right]}^{a}{\left[-\Phi^{-1}(\vartheta )\right]}^{b}.$$

These are characteristic coefficients describing the progress in the limit *μ*, λ → ∞ with constant *ϑ* = *μ*/λ, and are related to the generalized progress coefficients [5, Eq. (5.112)]. They will reappear during the derivation of both *φ_i_* and $\varphi_{i}^{\text{II}}$. The derivation of *φ_i_* is presented now.

### Proposition 1

*Let μ*,λ ∈ ℕ *with μ* ≥ 1 *and μ < λ and let p_x_ denote the PDF of the random mutation x* ~ 𝒩 (0,σ^2^). *Let x*_*m*;λ_ *denote the m-th best value (out of* λ*) of the i-th mutation component* (**x**_*m*;λ_)*_i_. Furthermore, let P_Q_ and* $P_{Q}^{-1}$ *denote the quality gain CDF (and its inverse), respectively, with* B *denoting the beta function. Then, the first order component-wise progress rate is given by*

(46) $$\begin{array}{l} \varphi_{i}=-\frac{1}{\mu }\sum_{m=1}^{\mu }\text{E}\left[x_{m;\text{λ}}\right]\\ \;=-\frac{\text{λ}}{\mu }\int_{x_{i}=-\infty }^{x_{i}=\infty }x_{i}p_{x}(x_{i})\frac{1}{\;\text{B}(\text{λ}-\mu ,\mu )}\int_{t=0}^{t=1}t^{\text{λ}-\mu -1}{\left(1-t\right)}^{\mu -1}P_{Q}(P_{Q}^{-1}(1-t)|x_{i})\text{d}t\;\text{d}x_{i}. \end{array}$$

### Proof

From now on the conditional dependency on **y**^(*g*)^ and *σ*^(*g*)^ will be implicitly assumed as given for better readability of the equations. The expected value of the *i*-th mutation component *x*_*m;*λ_ after selection can be expressed as an integral over the order statistic density *p*_*m*;λ_(*x_i_*) of the *m*-th best individual, such that (36) is rewritten as

(47) $$\varphi_{i}=-\frac{1}{\mu }\sum_{m=1}^{\mu }\text{E}\left[x_{m;\text{λ}}\right]=-\frac{1}{\mu }\sum_{m=1}^{\mu }\int_{-\infty }^{\infty }x_{i}p_{m;\text{λ}}\left(x_{i}\right)\text{d}x_{i}.$$

The subsequent task will be to derive the density *p*_*m*;λ_ as a function of mutation and quality gain distributions. Mutations are distributed normally with zero mean and variance *σ*^2^ according to the normal density

(48) $$p_{x}(x_{i})=\frac{1}{\sqrt{2\pi }\sigma }\exp\left[-\frac{1}{2}{\left(\frac{x_{i}}{\sigma }\right)}^{2}\right].$$

Given mutation *x_i_* (and implicitly position **y**), a random quality gain value *Q* is distributed according to a conditional probability density *p_Q_* (*q*|*x_i_*). Given that the *m*-th best individual attains a quality gain within [*q, q* + d*q*], there must be *m* − 1 better individuals having a smaller quality value with probability [Pr{*Q* ≤ *q*}]^*m*−1^ = [*P_Q_* (*q*)]^*m*−1^, and λ − *m* individuals having a larger value with [Pr{*Q* > *q*}]^λ−*m*^ = [1 − *P_Q_* (*q*)]^λ−*m*^. To account for all relevant combinations one has $\frac{\text{λ}!}{(m-1)!(\text{λ}-m)!}$, where 1/(*m* − 1)! and 1/(λ − *m*)! exclude the irrelevant combinations among the two groups of better and worse individuals, respectively. The conditional density for the *m*-th individual as a function of the quality gain *q* yields

(49) $$p_{Q;m;\text{λ}}\left(q|x_{i}\right)=\frac{\text{λ}!}{(m-1)!(\text{λ}-m)!}p_{Q}(q|x_{i})P_{Q}(q{)}^{m-1}[1-P_{Q}(q){]}^{\text{λ}-m}.$$

By integrating (49) over all attainable quality gain values *q* ∈ [*q_l_*, *q_u_*], one arrives at the density

(50) $$p_{m;\text{λ}}(x_{i})=p_{x}(x_{i})\frac{\text{λ}!}{(m-1)!(\text{λ}-m)!}\int_{q_{l}}^{q_{u}}p_{Q}(q|x_{i})P_{Q}{\left(q\right)}^{m-1}{\left[1-P_{Q}(q)\right]}^{\text{λ}-m}\;\text{d}q.$$

Inserting the order statistic density from (50) into the progress rate (47), one obtains the intermediate result

(51) $$\varphi_{i}=-\frac{1}{\mu }\sum_{m=1}^{\mu }\frac{\text{λ}!}{(m-1)!(\text{λ}-m)!}\int_{-\infty }^{\infty }x_{i}p_{x}(x_{i})\int_{q_{l}}^{q_{u}}p_{Q}(q|x_{i})P_{Q}{\left(q\right)}^{m-1}{\left[1-P_{Q}(q)\right]}^{\text{λ}-m}\;\text{d}q\;\text{d}x_{i}.$$

A few important remarks can be made regarding Eq. (51). A closed-form analytic solution cannot be obtained without applying further approximations. It can be approached in an analogous way to the *φ_i_*-derivation of the Ellipsoid in [13] to obtain a solution in terms of the well-known progress coefficient *c*_*μ/μ,*λ_ [5, p. 216]. However, a closed-form solution with this approach requires a linear relation of *Q_i_* w.r.t. *x_i_*, see relation (32). The effect of a linearized quality gain on the progress rate of the Rastrigin function was already studied in [17] and showed that the progress due to local attraction is not modeled correctly, as the oscillation terms have to be either dropped or linearized for small *x_i_*.

Therefore a different approach is followed here assuming the infinite population limit, an approach which was applied within the analysis of functions with noise-induced multi-modality [9]. The approach will yield correction terms including the effects of the trigonometric terms from (24), in contrast to only taking linearized terms from (32). Starting from Eq. (51) and moving the sum including the *m*-dependent prefactors into the innermost integral yields

(52) $$\varphi_{i}=-\frac{\text{λ}!}{\mu }\int_{-\infty }^{\infty }x_{i}p_{x}(x_{i})\int_{q_{l}}^{q_{u}}p_{Q}(q|x_{i})\sum_{m=1}^{\mu }\frac{P_{Q}{\left(q\right)}^{m-1}{\left[1-P_{Q}(q)\right]}^{\text{λ}-m}}{(m-1)!(\text{λ}-m)!}\text{d}q\;\text{d}x_{i}.$$

Now a transformation can be applied for the sum Σ_*m*_(·) yielding an expression as a function of the regularized incomplete beta function [5, p. 147]. One has

(53) $$\sum_{m=1}^{\mu }\frac{P{\left(q\right)}^{m-1}{\left[1-P(q)\right]}^{\text{λ}-m}}{(m-1)!(\text{λ}-m)!}=\frac{1}{(\text{λ}-\mu -1)!(\mu -1)!}\int_{0}^{1-P(q)}t^{\text{λ}-\mu -1}{\left(1-t\right)}^{\mu -1}\;\text{d}t.$$

Furthermore, one can rewrite the resulting population-dependent factor as follows

(54) $$\frac{\text{λ}!}{\mu }\frac{1}{(\text{λ}-\mu -1)!(\mu -1)!}=\frac{\text{λ}}{\mu }\frac{(\text{λ}-1)!}{(\text{λ}-\mu -1)!(\mu -1)!}=\frac{\text{λ}}{\mu }\frac{\Gamma (\text{λ})}{\Gamma (\text{λ}-\mu )\Gamma (\mu )}=\frac{\text{λ}}{\mu }\frac{1}{\;\text{B}(\text{λ}-\mu ,\mu )},$$

where we have used the property of the gamma function Γ(*n*) = (*n* − 1)! (for any integer *n* > 0) and the known relation between gamma and beta functions $\frac{\text{Γ}(x)\text{Γ}(y)}{\text{Γ}(x+y)}=\text{B}(x,y)$. These replacements will be useful later. After replacing the sum and refactoring we arrive at the following progress rate integral

(55) $$\varphi_{i}=-\frac{\text{λ}}{\mu }\frac{1}{\;\text{B}(\text{λ}-\mu ,\mu )}\int_{x_{i}=-\infty }^{x_{i}=\infty }x_{i}p_{x}(x_{i})\int_{q=q_{l}}^{q=q_{u}}p_{Q}\left(q|x_{i}\right)\int_{t=0}^{t=1-P_{Q}(q)}t^{\text{λ}-\mu -1}{\left(1-t\right)}^{\mu -1}\;\text{d}t\;\text{d}q\;\text{d}x_{i}.$$

Now the integration order of *t* and *q* is exchanged. In Eq. (55) one has the bounds

(56) $$q_{l}\leq q\leq q_{u},\;0\leq t\leq 1-P_{Q}(q).$$

Defining the inverse transformation $q=P_{Q}^{-1}(1-t)$ and integrating over *t* first, one obtains the new ranges

(57) $$0\leq t\leq 1,\;q_{l}\leq q\leq P_{Q}^{-1}(1-t).$$

The progress rate yields

(58) $$\varphi_{i}=-\frac{\text{λ}}{\mu }\frac{1}{\text{B}(\text{λ}-\mu ,\mu )}\int_{x_{i}=-\infty }^{x_{i}=\infty }x_{i}p_{x}\left(x_{i}\right)\int_{t=0}^{t=1}t^{\text{λ}-\mu -1}{\left(1-t\right)}^{\mu -1}\int_{q=q_{l}}^{q=P_{Q}^{-1}(1-t)}p_{Q}\left(q|x_{i}\right)\text{d}q\;\text{d}t\;\text{d}x_{i}.$$

Now the innermost integral can be solved using *p_Q_* (*q*|*x_i_*) = d*P_Q_* (*q*|*x_i_*)/d*q*

(59) $$\int_{q_{l}}^{P_{Q}^{-1}(1-t)}p_{Q}\left(q|x_{i}\right)\text{d}q=P_{Q}\left(P_{Q}^{-1}(1-t)|x_{i}\right)-P_{Q}\left(q_{l}|x_{i}\right)=P_{Q}\left(P_{Q}^{-1}(1-t)|x_{i}\right),$$

where the probability *P_Q_* (*q_l_*|*x_i_*) = Pr(*Q* ≤ *q_l_*|*x_i_*) = 0 for any lower bound value *q_l_*. Inserting (59) into (58), we arrive at the progress rate integral (46).

Unfortunately a closed-form solution of (46) after inserting Approximation 1 and Approximation 2 for the quality gain CDF is not possible due to the underlying structure of the integrand. Hence, asymptotic approximations will be introduced assuming large populations and large dimensionality to successively simplify the integral in a way that closed-form solutions can be provided. First, the large population theorem will be applied and then the quality gain CDF is inserted. Thereafter, the normal CDF is Taylor-expanded with the first two terms yielding analytically solvable results and higher order terms vanishing as *O*(1/*N*). The results are further simplified in the end assuming component equipartition (12), which finally gives the progress rate result in (96).

### Theorem 2

*Let p_x_ denote the PDF of the random mutation x* ~ 𝒩 (0, *σ*^2^). *Let P_Q_ denote the quality gain CDF with its quantile function given by* $P_{Q}^{-1}$. *For a truncation ratio ϑ = μ*/λ *with* 0 < *ϑ* < 1 *the component-wise progress rate for large populations yields*

(60) $$\underset{\begin{array}{c} \mu ,\text{λ}\to \infty \\ \vartheta =\text{ const}. \end{array}}{\lim}\varphi_{i}=-\frac{1}{\vartheta }\int_{-\infty }^{\infty }x_{i}p_{x}\left(x_{i}\right)P_{Q}\left(P_{Q}^{-1}(\vartheta )|x_{i}\right)\text{d}x_{i}.$$

### Proof

Starting from Eq. (46) and applying the infinite population size limit, the result of Theorem 1 can be applied with *a* = 1, *p_n_*(*x_i_*) = *x_i_*, and $f_{x}(t)=P_{Q}\left(P_{Q}^{-1}(1-t)|x_{i}\right)$. Evaluating *f_x_*(*t*) at *t* = 1 − *ϑ* gives

(61) $${f_{x}(t)|}_{t=1-\vartheta }={P_{Q}\left(P_{Q}^{-1}(1-t)|x_{i}\right)|}_{t=1-\vartheta }=P_{Q}\left(P_{Q}^{-1}(\vartheta )|x_{i}\right),$$

which yields the result (60).

The next step requires the use of Approximation 1 and Approximation 2 for the quality gain distributions in Eq. (60). To this end, one uses the conditional normal distribution function $\text{Φ}\left(\frac{q-E_{Q|x_{i}}}{D_{i}}\right)$, see (21), and the inverse transformation *q* = *E_Q_* + *D_Q_* Φ^−1^(*p*) evaluated at *p* = *ϑ*, see (18). One obtains

(62) $${\tilde{P}}_{Q}\left({\tilde{P}}_{Q}^{-1}(\vartheta )|x_{i}\right)=\text{Φ}\left(\frac{E_{Q}+D_{Q}{\text{Φ}}^{-1}(\vartheta )-E_{Q|x_{i}}}{D_{i}}\right).$$

Given the normal approximation (62), an expression for *E*_*Q*|*x_i_*_ is needed. Using definition (19) with *Q_i_*-result (24) the (conditional) expected value is written as

(63) $$E_{Q|x_{i}}=Q_{i}\left(x_{i}\right)+\underset{j\neq i}{\sum }\text{E}\left[Q_{j}\right]=k_{i}x_{i}+\delta_{i}\left(x_{i}\right)+E_{i}.$$

In (63) the following definitions are introduced as abbreviations

(64) $$\begin{array}{l} \;k_{i}:=2y_{i}\\ \delta_{i}\left(x_{i}\right):=x_{i}^{2}+A\cos\left(\alpha y_{i}\right)\left(1-\cos\left(\alpha x_{i}\right)\right)+A\sin\left(\alpha y_{i}\right)\sin\left(\alpha x_{i}\right)\\ \;E_{i}:=\underset{j\neq i}{\sum }\text{E}\left[Q_{i}\right]. \end{array}$$

Given Eq. (63), quantity *δ*(*x_i_*) includes all non-linear terms in *x_i_*. This will be important when the normal CDF is expanded and analytically solved. Inserting relation (63) into (62) and the result into (60) yields

(65) $$\varphi_{i}≃-\frac{1}{\vartheta }\int_{-\infty }^{\infty }x_{i}p_{x}\left(x_{i}\right)\text{Φ}\left(\frac{E_{Q}+D_{Q}{\text{Φ}}^{-1}(\vartheta )-\left(k_{i}x_{i}+\delta_{i}\left(x_{i}\right)+E_{i}\right)}{D_{i}}\right)\text{d}x_{i}.$$

A closed-form solution of (65) cannot be obtained with Φ(*δ_i_*(*x_i_*)) containing non-linear terms in *x_i_*. However, a solution in terms of a Taylor expansion can be provided by introducing the decomposition Φ(*g*(*x_i_*) + *h*(*x_i_*)) with *g*(*x_i_*) being a linear function, and *h*(*x_i_*) being a small non-linear perturbation according to

(66) $$g\left(x_{i}\right):=-\frac{k_{i}}{D_{i}}x_{i}+\frac{E_{Q_{i}}+D_{Q}{\text{Φ}}^{-1}(\vartheta )}{D_{i}}$$

(67) $$h\left(x_{i}\right):=-\frac{\delta \left(x_{i}\right)}{D_{i}}.$$

In (66), the abbreviation *E_Q_i__* = *E_Q_* − *E_i_* = E[*Q_i_*], cf. Eq. (10), is used to denote the expected value of the *i*-th summand of the quality gain (6). Using functions *g*(*x_i_*) and *h*(*x_i_*) Eq. (65) becomes

(68) $$\varphi_{i}≃-\frac{1}{\vartheta }\int_{-\infty }^{\infty }x_{i}p_{x}\left(x_{i}\right)\text{Φ}\left(g\left(x_{i}\right)+h\left(x_{i}\right)\right)\text{d}x_{i}.$$

### Approximation 3 *(Truncated cumulative distribution function series)*

Under the assumption of a normally distributed quality gain, see Approximation 1 and Approximation 2, and a quality gain variance scaling with *N* according to Eq. (13), the CDF of the normal distribution is expanded at *g*(*x_i_*) in the limit of *N* → ∞ as

(69) $$\varphi_{i}≃-\frac{1}{\vartheta }\int_{-\infty }^{\infty }x_{i}p_{x}\left(x_{i}\right)\left(\text{Φ}\left(g\left(x_{i}\right)\right)+\phi \left(g\left(x_{i}\right)\right)h\left(x_{i}\right)+O\left(\frac{1}{N}\right)\right)\text{d}x_{i}.$$

Relation (69) is derived now. Starting from (68), the Taylor-expansion of Φ(·) up to first order with the remainder denoted by *r* yields

(70) $$\text{Φ}(g+h)=\overset{\infty }{\underset{n=0}{\sum }}\frac{1}{n!}\frac{{\text{d}}^{n}\text{Φ}}{\text{d}g^{n}}h^{n}=\text{Φ}(g)+\phi (g)h+r(N).$$

Note that all derivatives of the normal distribution exist as $\frac{{\text{d}}^{n}\phi (x)}{\text{d}x^{n}}=(-1{)}^{n}{\text{He}}_{n}(x)\phi (x)$ with He*_n_* (*x*) denoting the *n*-th order probabilist’s Hermite polynomials. In the following the scaling properties of the remainder as a function of *N* are investigated. It will be shown that *r* = *O*(1/*N*). To this end, (70) is rewritten as

(71) r(N)=Φ(g+h)−Φ(g)−ϕ(g)h.

For the further analysis of *r*(*N*) the equipartition of components is assumed as introduced in Eqs. (12), (13), and (14). Hence, the variance *D_i_* can be written as a function of *N* as

(72) $$D_{i}=s\sqrt{N},$$

where the prefactor *s* ≠ *s*(*N*) depends on *A*, *α*, **y**, and *σ*. With these assumptions the functions *g* and *h* are written as (using *E* := *E_Q_i__*, ${\text{Φ}}_{\vartheta }^{-1}:={\text{Φ}}^{-1}(\vartheta )$, dropping the subscript *i* for brevity and using *D_i_* ≃ *D_Q_*)

(73) $$g=\frac{E-kx}{s\sqrt{N}}+{\text{Φ}}_{\vartheta }^{-1},\;h=-\frac{\delta }{s\sqrt{N}}.$$

As *h* → 0 for *N* → ∞, the remainder (71) vanishes accordingly. Therefore, in order to show *r*(*N*) = *O*(1/*N*), lim_*N*→∞_ *r*(*N*)*N* is investigated applying l’Hôpital’s rule

(74) $$\underset{N\to \infty }{\lim}r(N)N=\underset{N\to \infty }{\lim}\frac{r(N)}{1/N}=\underset{N\to \infty }{\lim}\frac{\frac{\partial r(N)}{\partial N}}{\frac{\partial (1/N)}{\partial N}}=-\underset{N\to \infty }{\lim}N^{2}\frac{\partial r(N)}{\partial N}.$$

To evaluate (74) the derivative of *r* from (71) w.r.t. *N* is evaluated as

(75) $$\begin{array}{lll} \frac{\partial r}{\partial N} & = & \frac{1}{\sqrt{2\pi }}{\text{e}}^{-\frac{1}{2}{\left(g+h\right)}^{2}}\left(\frac{\partial g}{\partial N}+\frac{\partial h}{\partial N}\right)-\frac{1}{\sqrt{2\pi }}{\text{e}}^{-\frac{1}{2}g^{2}}\frac{\partial g}{\partial N}+\frac{gh}{\sqrt{2\pi }}{\text{e}}^{-\frac{1}{2}g^{2}}\frac{\partial g}{\partial N}-\frac{1}{\sqrt{2\pi }}{\text{e}}^{-\frac{1}{2}g^{2}}\frac{\partial h}{\partial N}\\ & = & \frac{1}{\sqrt{2\pi }}{\text{e}}^{-\frac{1}{2}g^{2}}\left[\left({\text{e}}^{-gh-\frac{1}{2}h^{2}}-1\right)\left(\frac{\partial g}{\partial N}+\frac{\partial h}{\partial N}\right)+gh\frac{\partial g}{\partial N}\right]. \end{array}$$

The term $\left({\text{e}}^{-gh-\frac{1}{2}h^{2}}-1\right)$ of (75) is expanded up to first order discarding higher orders $O\left({\left(gh+\frac{1}{2}h^{2}\right)}^{2}\right)$

(76) $$\begin{array}{lll} \frac{\partial r}{\partial N} & ≃ & \frac{1}{\sqrt{2\pi }}{\text{e}}^{-\frac{1}{2}g^{2}}\left[\left(-gh-\frac{1}{2}h^{2}\right)\left(\frac{\partial g}{\partial N}+\frac{\partial h}{\partial N}\right)+gh\frac{\partial g}{\partial N}\right]\\ & = & \frac{1}{\sqrt{2\pi }}{\text{e}}^{-\frac{1}{2}g^{2}}\left[-\frac{1}{2}h^{2}\left(\frac{\partial g}{\partial N}+\frac{\partial h}{\partial N}\right)-gh\frac{\partial h}{\partial N}\right]. \end{array}$$

The derivatives of *g* and *h* from Eq. (73) are

(77) $$\frac{\partial g}{\partial N}=-\frac{E-kx}{2sN^{3/2}},\;\frac{\partial h}{\partial N}=\frac{\delta }{2sN^{3/2}}.$$

Inserting (73) and (77) into (76) yields after refactoring

(78) $$\begin{array}{lll} \frac{\partial r}{\partial N} & ≃ & \frac{1}{\sqrt{2\pi }}{\text{e}}^{-\frac{1}{2}{\left(\frac{E-kx}{s\sqrt{N}}+{\text{Φ}}_{\vartheta }^{-1}\right)}^{2}}\left[-\frac{\delta^{2}}{2s^{2}N}\left(-\frac{E-kx}{2sN^{3/2}}+\frac{\delta }{2sN^{3/2}}\right)+\left(\frac{E-kx}{s\sqrt{N}}+{\text{Φ}}_{\vartheta }^{-1}\right)\frac{\delta^{2}}{2s^{2}N^{2}}\right]\\ & = & \frac{1}{\sqrt{2\pi }}{\text{e}}^{-\frac{1}{2}{\left(\frac{E-kx}{s\sqrt{N}}+{\text{Φ}}_{\vartheta }^{-1}\right)}^{2}}\left(-\frac{\delta^{2}}{2s^{2}N^{2}}\right)\left[\frac{\delta }{2s\sqrt{N}}-\frac{3}{2}\frac{E-kx}{s\sqrt{N}}-{\text{Φ}}_{\vartheta }^{-1}\right]. \end{array}$$

Taking the limit (74) of (78) therefore yields

(79) $$\begin{array}{lll} \underset{N\to \infty }{\lim}r(N)N=-\underset{N\to \infty }{\lim}N^{2}\frac{\partial r(N)}{\partial N} & = & \underset{N\to \infty }{\lim}\left\{\frac{1}{\sqrt{2\pi }}{\text{e}}^{-\frac{1}{2}{\left(\frac{E-kx}{s\sqrt{N}}+{\text{Φ}}_{\vartheta }^{-1}\right)}^{2}}\frac{\delta^{2}}{2s^{2}}\left[\frac{\delta }{2s\sqrt{N}}-\frac{3}{2}\frac{E-kx}{s\sqrt{N}}-{\text{Φ}}_{\vartheta }^{-1}\right]\right\}\\ & = & -\frac{\delta^{2}{\text{Φ}}_{\vartheta }^{-1}}{2\sqrt{2\pi }s^{2}}{\text{e}}^{-\frac{1}{2}{\left({\text{Φ}}_{\vartheta }^{-1}\right)}^{2}}, \end{array}$$

such that the remainder *r*(*N*) can be given as

(80) $$r(N)≃-\frac{\delta^{2}{\text{Φ}}_{\vartheta }^{-1}}{2\sqrt{2\pi }s^{2}}{\text{e}}^{-\frac{1}{2}{\left({\text{Φ}}_{\vartheta }^{-1}\right)}^{2}}\frac{1}{N}=O(\frac{1}{N}),$$

which concludes the derivation of (69).

Both integrals of (69) are analytically solvable.^2^ The zeroth order term yields a closed form solution due to *g*(*x_i_*) being linear w.r.t. *x_i_* and gives progress contributions due to the sphere function, i.e., the linear part of the quality gain (63). The first order term can be solved by applying quadratic completion to the Gaussian product *p_x_*(*x_i_*)*ϕ*(*g*(*x_i_*)) yielding an expected value over a normal density. The expected value over *h*(*x_i_*) can be regarded as a perturbation of the sphere containing *A* and *α* dependencies.

The determination of *φ_i_* via (69) was done in [18] by evaluating both integrals. As the derivation and the final result for *φ_i_* are very lengthy and therefore not practical for further analytic treatment, the obtained expression for *φ_i_* was simplified as a last step assuming large dimensionality *N*. However, the same result as in [18] can be obtained in a quicker way by simplifying the integrands of (69) under the same assumptions before the integration, instead of simplifying the result afterwards. This will enable a more concise derivation of the final progress rate result.

First the functions *g* and *h* from (66) and (67), respectively, are simplified. For large *N*, the quality gain variance *D_i_* ≃ *D_Q_* using (14). As *E_Q_i__* is just the quality gain expectation of a single component, it can be neglected compared to *D_Q_* scaling as $\sqrt{N}$ using (13). Hence, one has

(81) $$g\left(x_{i}\right)≃-\frac{k_{i}x_{i}}{D_{Q}}+{\text{Φ}}^{-1}(\vartheta )$$

(82) $$h\left(x_{i}\right)≃-\frac{\delta \left(x_{i}\right)}{D_{Q}}.$$

Another approximation is introduced regarding the density *p_x_*(*x_i_*)*ϕ*(*g*(*x_i_*)) for the second term of (69). By completing the square one can derive a resulting normal density with mean *m* and variance *ς*^2^ by demanding

(83) $$p_{x}\left(x_{i}\right)\phi \left(g\left(x_{i}\right)\right)=\frac{1}{\sqrt{2\pi }\sigma }{\text{e}}^{-\frac{1}{2}\frac{x_{i}^{2}}{\sigma^{2}}}\frac{1}{\sqrt{2\pi }}{\text{e}}^{-\frac{1}{2}g{\left(x_{i}\right)}^{2}}\overset{!}{=}C{\text{e}}^{-\frac{1}{2}\frac{{\left(x_{i}-m\right)}^{2}}{\varsigma^{2}}}.$$

Simple calculations yield

(84) $$m={\text{Φ}}^{-1}(\vartheta )\frac{D_{Q}k_{i}\sigma^{2}}{D_{Q}^{2}+k_{i}^{2}\sigma^{2}},\;\varsigma^{2}=\frac{1}{1/\sigma^{2}+\left(k_{i}^{2}/D_{Q}^{2}\right)},\;C=\frac{{\text{e}}^{-\frac{1}{2}{\left[{\text{Φ}}^{-1}(\vartheta )\right]}^{2}}}{2\pi \sigma }.$$

Noting that $D_{Q}^{2}=\text{Θ}(N)$ and neglecting contributions of single components for *N* → ∞, i.e., $k_{i}^{2}\ll D_{Q}^{2}$, ${\left(k_{i}\sigma \right)}^{2}\ll D_{Q}^{2}$, the quantities *m* and *ς*^2^ from (84) yield the asymptotic results

(85) $$m≃0,\;\varsigma^{2}≃\sigma^{2},$$

such that the density of the first order term yields

(86) $$p_{x}\left(x_{i}\right)\phi \left(g\left(x_{i}\right)\right)≃\frac{{\text{e}}^{-\frac{1}{2}{\left[{\text{Φ}}^{-1}(\vartheta )\right]}^{2}}}{\sqrt{2\pi }}p_{x}\left(x_{i}\right).$$

Using the results from Eqs. (81), (82), and (86), the progress rate integral (69) is further simplified. The prefactors of the resulting integral yield the asymptotic progress coefficient (45)

(87) $$c_{\vartheta }:=e_{\vartheta }^{1,0}=\frac{1}{\sqrt{2\pi }\vartheta }{\text{e}}^{-\frac{1}{2}{\left[{\text{Φ}}^{-1}(\vartheta )\right]}^{2}}.$$

### Approximation 4 *(Progress rate integral for large dimensionality)*

Based on the result of Approximation 3 only the first two terms are considered. Furthermore, the integrands of (69) are approximated and simplified assuming large dimensionality using Eqs. (81), (82), (86), and (87). Hence, one obtains

(88) $$\varphi_{i}≃I_{i}^{0}+I_{i}^{1},\;\text{with}$$

(89) $$I_{i}^{0}:=-\frac{1}{\vartheta }\int_{-\infty }^{\infty }x_{i}p_{x}\left(x_{i}\right)\text{Φ}\left(-\frac{k_{i}x_{i}}{D_{Q}}+{\text{Φ}}^{-1}(\vartheta )\right)\text{d}x_{i},\;\text{and}$$

(90) $$I_{i}^{1}:=\frac{c_{\vartheta }}{D_{Q}}\frac{1}{\sqrt{2\pi }\sigma }\int_{-\infty }^{\infty }x_{i}\delta \left(x_{i}\right)p_{x}\left(x_{i}\right)\text{d}x_{i}.$$

Calculating $I_{i}^{0}$ from (89) by inserting mutation density *p_x_*(*x_i_*) from (48) and applying the substitution *z* = *x_i_*/*σ*, one gets

(91) $$I_{i}^{0}=-\frac{\sigma }{\sqrt{2\pi }\vartheta }\int_{-\infty }^{\infty }z{\text{e}}^{-\frac{1}{2}z^{2}}\text{Φ}\left(-\frac{k_{i}\sigma }{D_{Q}}z+{\text{Φ}}^{-1}(\vartheta )\right)\text{d}z.$$

The following integral identity [5, Eq. (A.12)] can be applied

(92) $$\int_{-\infty }^{\infty }t{\text{e}}^{-\frac{1}{2}t^{2}}\text{Φ}(at+b)\text{d}t=\frac{a}{\sqrt{1+a^{2}}}\exp\left[-\frac{1}{2}\frac{b^{2}}{1+a^{2}}\right].$$

Evaluating (92) with *a* = −*k_i_σ/D_Q_* and *b* = Φ^−1^(*ϑ*) yields for the right-hand side of (92)

(93) $$\frac{a}{\sqrt{1+a^{2}}}\exp\left[-\frac{1}{2}\frac{b^{2}}{1+a^{2}}\right]=-\frac{k_{i}\sigma }{D_{Q}}\frac{1}{\sqrt{1+{\left(k_{i}\sigma /D_{Q}\right)}^{2}}}\exp[-\frac{1}{2}\frac{{\left[{\text{Φ}}^{-1}(\vartheta )\right]}^{2}}{1+{\left(k_{i}\sigma /D_{Q}\right)}^{2}}].$$

Again assuming ${\left(k_{i}\sigma \right)}^{2}\ll D_{Q}^{2}$, expression (93) simplifies and the result for (89) is obtained with (87) as

(94) $$I_{i}^{0}≃\frac{{\text{e}}^{-\frac{1}{2}{\left[{\text{Φ}}^{-1}(\vartheta )\right]}^{2}}}{\sqrt{2\pi }\vartheta }\frac{k_{i}\sigma^{2}}{D_{Q}}=c_{\vartheta }\frac{k_{i}\sigma^{2}}{D_{Q}}.$$

Now $I_{i}^{1}$ is solved. One notices that $x_{i}\delta (x_{i})=x_{i}(x_{i}^{2}+A\;\text{cos}\;(\alpha y_{i})(1-\text{cos}\;(\alpha x_{i}))+A\;\text{sin}\;(\alpha y_{i})\text{sin}\;(\alpha x_{i})),$ see (64), is integrated over density *p_x_* with zero mean. Therefore, all odd functions of *x_i_* yield no contribution and only the term *x_i_* sin (*αx_i_*) needs to be evaluated. One gets

(95) $$\begin{array}{lll} I_{i}^{1} & ≃ & c_{\vartheta }\frac{A\sin\left(\alpha y_{i}\right)}{D_{Q}}\frac{1}{\sqrt{2\pi }\sigma }\int_{-\infty }^{\infty }x_{i}\sin\left(\alpha x_{i}\right){\text{e}}^{-\frac{1}{2}{\left(\frac{x_{i}}{\sigma }\right)}^{2}}\text{d}x_{i}\\ & = & c_{\vartheta }\frac{A\sin\left(\alpha y_{i}\right)}{D_{Q}}\text{E}\left[x_{i}\sin\left(\alpha x_{i}\right)\right]\\ & = & c_{\vartheta }\frac{A\sin\left(\alpha y_{i}\right)}{D_{Q}}\alpha \sigma^{2}{\text{e}}^{-\frac{1}{2}{\left(\alpha \sigma \right)}^{2}}\\ & = & c_{\vartheta }\frac{d_{i}\sigma^{2}}{D_{Q}}e^{-\frac{1}{2}{\left(\alpha \sigma \right)}^{2}}. \end{array}$$

In the second line of (95) the expected value definition is used. From second to third line the expected value of *x_i_* sin (*αx_i_*) is evaluated using (29). In the last line the derivative *d_i_* = *αA* sin (*αy_i_*) from (33) is recovered. Using the results from (94) and (95) the first order progress rate approximation for large *N* and *μ* can finally be given.

#### First order progress rate

*The first order component-wise progress rate on the Rastrigin function in the asymptotic limits of infinitely large population size μ (constant ϑ = μ*/λ*) and infinitely large dimensionality N yields*

(96) $$\varphi_{i}≃c_{\vartheta }\frac{\sigma^{2}}{D_{Q}}\left(k_{i}+{\text{e}}^{-\frac{1}{2}{\left(\alpha \sigma \right)}^{2}}d_{i}\right)=c_{\vartheta }\frac{\sigma^{2}}{D_{Q}}\left(2y_{i}+{\text{e}}^{-\frac{1}{2}{\left(\alpha \sigma \right)}^{2}}\alpha A\sin\left(\alpha y_{i}\right)\right).$$

The expressions for $c_{\vartheta }=e_{\vartheta }^{1,0}$ from (45) and *D_Q_* from (31) were not inserted to improve readability. Result (96) shows very interesting properties compared to [17, Eq. (26)], where a linearized quality gain approximation resulted in

(97) $$\varphi_{i,\text{ lin }}≃c_{\mu /\mu ,\text{λ}}\frac{\sigma^{2}}{\sqrt{{\left(f_{i}^{\prime }\sigma \right)}^{2}+D_{i}^{2}}}f_{i}^{\prime }.$$

First note that the progress coefficient was replaced by its asymptotic form *c*_*μ/μ,*λ_ ≃ *c_ϑ_*. The difference for the variance terms in the denominators of (96) and (97) is negligible for large *N* with $D_{Q}^{2}\approx D_{i}^{2}+{\left(f_{i}^{\prime }\sigma \right)}^{2}$, see also (14). However, the most notable difference lies between the derivative term $f_{i}^{\prime }=k_{i}+d_{i}$, see definition (33), and the newly obtained term $k_{i}+{\text{e}}^{-\frac{1}{2}{\left(\alpha \sigma \right)}^{2}}d_{i}$. It contains an unchanged sphere-dependent term *k_i_* and an exponentially decaying Rastrigin-specific term *d_i_*. This characteristic form will be discussed in the subsequent part. The result (96) will be essential for the determination of the second order $\varphi_{i}^{\text{II}}$.

At this point one-generation experiments can be performed and compared to the progress rate (96) to investigate its accuracy. To this end, a random position vector **y** is initialized isotropically with ‖**y**‖ = *R* given some residual distance *R*. Then, repeated simulations are performed and quantity (7) is averaged over 10^6^ trials. The issue with the choice of *R* is that the “interesting” region with high density of local minima scales with *N*, such that a relation *R*(*N*) is needed. The following argumentation can be given. Assuming w.l.o.g. **y** > **0** and that all components of the parental position are at some given local minimum denoted by ŷ^(*j*)^. Index *j* identifies the local attractor along the half-axis, e.g. *j* ∈ {1, 2, 3} in Fig. 1 on the right side. For *N* = 1 one has **y** = [*ŷ*^(*j*)^] and therefore *R*^2^ = (*ŷ*^(*j*)^)^2^. Having *N* components at the same *j*-th local minimum yields **y** = [*ŷ*^(*j*)^, *ŷ*^(*j*)^, …, *ŷ*^(*j*)^], such that *R*^2^ = *N*(*ŷ*^(*j*)^)^2^. A scaling $R=O(\sqrt{N})$ is therefore needed to stay within a certain region of local attractors when *N* is increased.

**Fig. 3:**
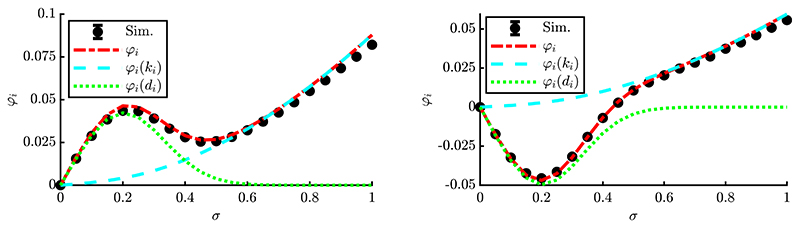
One-generation experiments with (10/10, 40)-ES for *N* = 20, *A* = 10, *α* = 2*π* at randomly chosen $\left\|\text{y}\right\|=R=\sqrt{N}.$ The results for *φ_i_* of Eq. (96) are shown for the exemplary components *i* = 2 with *y_i_* = 1.16 (left) and *i* = 12 with *y_i_* = 0.78 (right) to illustrate the effect of local attraction on the progress rate. The plots show additionally Eq. (96) with *φ_i_*(*k_i_*) = *φ_i_*(*d_i_*, *k_i_*)|_*d_i_*=0_ [cyan, dashed] and *φ_i_*(*d_i_*) = *φ_i_*(*d_i_*, *k_i_*)|_*k_i_*=0_ [green, dotted], respectively.

The progress rates of two exemplary components for a single experiment are shown in Fig. 3. For both plots *σ* ∈ [0, 1] was chosen in order to investigate the effects of the oscillation as *α* = 2*π*. On the left, one observes enhanced progress for moderate *σ*-values due to local attraction, as both local and global attractor are aligned along the same direction. On the right, there is negative progress for moderate *σ*, as the local attractor is driving the ES away from the global attractor. For larger *σ*, the overall spherical shape is dominating and both exhibit positive progress. A decomposition of the progress rate in terms of *φ_i_* = *φ_i_*(*d_i_*, *k_i_*)|_*k_i_*=0_ + *φ_i_*(*d_i_*, *k_i_*)|_*d_i_*=0_ is displayed in Fig. 3. It shows the large-scale behavior of the *k_i_*-term, dashed cyan, and limited range of the *d_i_*-term, dotted green. As $k_{i}=\partial \left(y_{i}^{2}\right)/\partial y_{i}$, its progress term models the global quadratic structure of Rastrigin, see derivative definitions (33). The second term ${\text{e}}^{-\frac{1}{2}{\left(\alpha \sigma \right)}^{2}}d_{i}$ models the Rastrigin-specific local oscillation having limited range depending on the mutation strength *σ* (or *α*). By defining scale-invariant mutations using (4) with *σ* = σ*R/N, the oscillations vanish via ${\text{e}}^{-\frac{1}{2}{\left(\alpha \sigma^{*}R/N\right)}^{2}}$ for large residual distance *R*, where the sphere function is recovered. This model significantly improves the progress rate formula (97) from [17].

As a note, changing one of the fitness parameters *A* or *α* directly affects Fig. 3. The change of amplitude *A* rescales both the (local) peak and dip heights accordingly, increasing the effects of local attraction for larger *A*. Increasing frequency *α* has mostly short-range effects as the overall range is reduced due to suppression via ${\text{e}}^{-\frac{1}{2}{\left(\alpha \sigma \right)}^{2}}$ of (96). In the subsequent parts, the progress rate is investigated for *A* = 1 and *α* = 2*π* as an example.

**Fig. 4:**
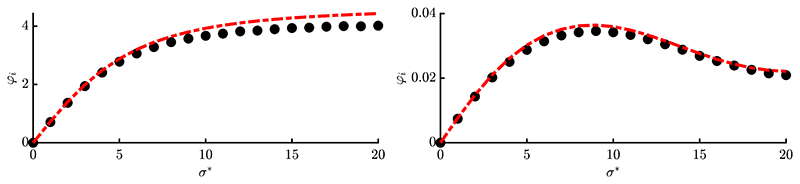
Progress rate *φ_i_* as a function of the normalized mutation σ* for (10/10, 40)-ES with *N* = 20, *A* = 1, *α* = 2*π*, at two residual distances $R=10\sqrt{N}$ with *y_i_* = 11.6 (left) and $R=0.1\sqrt{N}$ with *y_i_* = 0.116 (right). As in Fig. 3, black dots depict the simulation, while the red dash-dotted line shows result (96). The error bars are very small and therefore not visible.

**Fig. 5:**
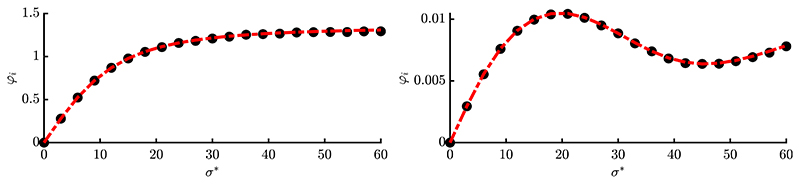
Progress rate *φ_i_* as a function of the normalized mutation σ* for (100/100, 200)-ES with *N* = 100, *A* = 1, *α* = 2*π*, at two residual distances $R=10\sqrt{N}$ with *y_i_* = 11.9 (left) and $R=0.1\sqrt{N}$ with *y_i_* = 0.119 (right). The approximation quality improves compared to Fig. 4 and shows very good agreement.

In Figs. 4 and 5 the progress rate is evaluated over scale-invariant σ* for two different *N*-values and population sizes. One can see that the approximation quality improves for larger *N* and *μ*, as expected from the applied approximations. The overall agreement between simulation and approximation is good for larger and smaller residual distances *R*, see left and right plots, respectively. The σ*-range was chosen large enough, such that the progress rate of the corresponding sphere function [5, Eq. (6.54)] reaches negative values due to mutations being too large. This boundary directly translates to Rastrigin, as the global structure is the same. However, due to *φ_i_* being first order, no negative progress occurs even for large σ*. Therefore the second order progress rate $\varphi_{i}^{\mathrm{II}}$ needs to be derived in Sec. 4, where loss terms will provide additional correction terms.

## 4 Second order progress rate

The second order progress rate (8) requires the evaluation of $\text{E}\left[{\left(y_{i}^{\left(g+1\right)}\right)}^{2}\right].$ Starting with intermediate result (34) and referring to the *i*-th component, the expression yields after squaring

(98) $$\begin{array}{lll} {\left(y_{i}^{\left(g+1\right)}\right)}^{2} & = & {\left(y_{i}^{\left(g\right)}+\frac{1}{\mu }\overset{\mu }{\underset{m=1}{\sum }}x_{m;\text{λ}}\right)}^{2}\\ & = & {\left(y_{i}^{\left(g\right)}\right)}^{2}+2y_{i}^{\left(g\right)}\frac{1}{\mu }\overset{\mu }{\underset{m=1}{\sum }}x_{m;\text{λ}}+\frac{1}{\mu^{2}}{\left(\overset{\mu }{\underset{m=1}{\sum }}x_{m;\text{λ}}\right)}^{2}. \end{array}$$

Squaring the last term can be evaluated by separating the sum into equal and unequal indices

(99) $$\begin{array}{lll} {\left(\overset{\mu }{\underset{m=1}{\sum }}x_{m;\text{λ}}\right)}^{2} & = & \left(\overset{\mu }{\underset{k=1}{\sum }}x_{k;\text{λ}}\right)\left(\overset{\mu }{\underset{l=1}{\sum }}x_{l;\text{λ}}\right)=\overset{\mu }{\underset{m=1}{\sum }}{\left(x_{m;\text{λ}}\right)}^{2}+\underset{k\neq l}{\sum }x_{k;\text{λ}}x_{l;\text{λ}}\\ & = & \overset{\mu }{\underset{m=1}{\sum }}{\left(x_{m;\text{λ}}\right)}^{2}+2\overset{\mu }{\underset{l=2}{\sum }}\overset{l-1}{\underset{k=1}{\sum }}x_{k;\text{λ}}x_{l;\text{λ}}. \end{array}$$

Inserting (99) into (98) and taking the expected value (conditional variables **y**^(*g*)^ and *σ*^(*g*)^ are implicitly assumed to be given) yields

(100) $$\text{E}\left[{\left(y_{i}^{\left(g+1\right)}\right)}^{2}\right]={\left(y_{i}^{\left(g\right)}\right)}^{2}+2y_{i}^{\left(g\right)}\frac{1}{\mu }\overset{\mu }{\underset{m=1}{\sum }}\text{E}\left[x_{m;\text{λ}}\right]+\frac{1}{\mu^{2}}\overset{\mu }{\underset{m=1}{\sum }}\text{E}\left[{\left(x_{m;\text{λ}}\right)}^{2}\right]+\frac{2}{\mu^{2}}\overset{\mu }{\underset{l=2}{\sum }}\overset{l-1}{\underset{k=1}{\sum }}\text{E}[x_{k;\text{λ}}x_{l;\text{λ}}].$$

Noting that $\varphi_{i}=-\frac{1}{\mu }\sum_{m=1}^{\mu }\text{E}\left[x_{m;\text{λ}}\right],$ see Eq. (36), and using (100) in $\varphi_{i}^{\text{II}}$-definition (8) yields the second order *i*-th component progress rate

(101) $$\varphi_{i}^{\text{II}}=2y_{i}^{\left(g\right)}\varphi_{i}-\frac{1}{\mu^{2}}E^{\left(2\right)}-\frac{2}{\mu^{2}}E^{\left(1,1\right)},$$

for which the two following expected values need to be determined

(102) $$\frac{1}{\mu^{2}}E^{\left(2\right)}:=\frac{1}{\mu^{2}}\overset{\mu }{\underset{m=1}{\sum }}\text{E}\left[{\left(x_{m;\text{λ}}\right)}^{2}\right]$$

(103) $$\frac{1}{\mu^{2}}E^{\left(1,1\right)}:=\frac{1}{\mu^{2}}\overset{\mu }{\underset{l=2}{\sum }}\overset{l-1}{\underset{k=1}{\sum }}\text{E}\left[x_{k;\text{λ}}x_{l;\text{λ}}\right].$$

In the subsequent parts the solutions to Eqs. (102) and (103) will be derived. Starting with (102), the solution requires order statistic density (50) for the *m*-th individual, large population identity (37), and the expansion of the normal CDF (69) up to first order. The resulting two integrals can then be solved analytically for large *N* and the results will simplify significantly.

### Proposition 2

*Let μ,*λ ∈ ℕ *with μ ≥ 1 and μ <* λ *and let p_x_ denote the PDF of the random mutation x ~* 𝒩 *(0, σ^2^). Let x*_*m;*λ_ *denote the m-th best value (out of* λ*) of the i-th mutation component (***x**_*m;*λ_*)_i_. Furthermore, let P_Q_ and* $P_{Q}^{-\text{1}}$ *denote the quality gain CDF (and its inverse), respectively, with* B *denoting the beta function. Then, the second order expected value reads*

(104) $$\frac{1}{\mu }\overset{\mu }{\underset{m=1}{\sum }}\text{E}\left[{\left(x_{m;\text{λ}}\right)}^{2}\right]=\frac{\text{λ}}{\mu }\int_{x_{i}=-\infty }^{x_{i}=\infty }x_{i}^{2}p_{x}\left(x_{i}\right)\frac{1}{\text{B}(\text{λ}-\mu ,\mu )}\overset{t=1}{\underset{t=0}{\int }}t^{\text{λ}-\mu -1}(1-t{)}^{\mu -1}P_{Q}\left(P_{Q}^{-1}(1-t)|x_{i}\right)\text{d}t\;\text{d}x_{i}.$$

### Proof

Starting from (102) and rewriting the expected value as an integral over order statistic density *p*_*m*;λ_(*x_i_*) yields

(105) $$\frac{1}{\mu }\overset{\mu }{\underset{m=1}{\sum }}\text{E}\left[{\left(x_{m;\text{λ}}\right)}^{2}\right]=\frac{1}{\mu }\overset{\mu }{\underset{m=1}{\sum }}\int_{-\infty }^{\infty }x_{i}^{2}p_{m;\text{λ}}\left(x_{i}\right)\text{d}x_{i}.$$

Both (47) and (105) have the same structure after inserting *p*_*m*;λ_(*x_i_*) from (50) and the integration over the squared mutation component is performed as the last step. The same steps as presented in the proof of Proposition 1 can therefore be applied with squared quantity $x_{i}^{2}$, which directly gives the result (104).

Analogously to the derivation of the first order progress rate in Sec. 3, a closed-form solution for (104) can only be provided by first applying the limit of large populations and then introducing approximations assuming large dimensionality *N*.

### Theorem 3

*Let p_x_ denote the PDF of the random mutation x ~ 𝒩 (0, σ^2^) and let x*_*m*;λ_ *denote the m-th best value (out of* λ*) of the i-th mutation component* (**x**_*m*;λ_*)_i_. Let P_Q_ denote the quality gain CDF with its quantile function given by $P_{Q}^{-1}$. For a truncation ratio ϑ the limit of the second order expected value reads*

(106) $$\underset{\begin{array}{c} \mu ,\text{λ}\to \infty \\ \vartheta =\text{ const}. \end{array}}{\lim}\frac{1}{\mu }\overset{\mu }{\underset{m=1}{\sum }}\text{E}\left[{\left(x_{m;\text{λ}}\right)}^{2}\right]=\frac{1}{\vartheta }\int_{-\infty }^{\infty }x_{i}^{2}p_{x}\left(x_{i}\right)P_{Q}\left(P_{Q}^{-1}(\vartheta )|x_{i}\right)\text{d}x_{i}.$$

### Proof

Starting from Eq. (104) and applying the infinite population size limit, the result of Theorem 1 can be applied with *a* = 1, $p_{n}(x_{i})=x_{i}^{2},\;\text{and}\;f_{x}(t){|}_{t=1-\vartheta }=P_{Q}(P_{Q}^{-1}(\vartheta )|x_{i}),$ which yields the result (106).

Given result (106), approximations are again applied to provide closed-form solutions. Inserting quality gain Approximation 1 and Approximation 2 via Eq. (62) into (106) leads (again) to an analytically not solvable integral due to non-linear terms in *x_i_* within Φ(·). Therefore, the CDF is expanded using Approximation 3 neglecting higher order terms *O*(1/*N*). Finally, the integrands are simplified assuming large dimensionality using Approximation 4. The result is therefore given after inserting *g*(*x_i_*) and *h*(*x_i_*) from (81) and (82) as

(107) $$\frac{1}{\mu^{2}}E^{\left(2\right)}≃I_{i}^{0}+I_{i}^{1},\;\text{with}$$

(108) $$I_{i}^{0}:=\frac{1}{\mu \vartheta }\int_{-\infty }^{\infty }x_{i}^{2}p_{x}\left(x_{i}\right)\text{Φ}\left(-\frac{k_{i}x_{i}}{D_{Q}}+{\text{Φ}}^{-1}(\vartheta )\right)\text{d}x_{i},\;\text{and}$$

(109) $$I_{i}^{1}:=-\frac{c_{\vartheta }}{\mu D_{Q}}\frac{1}{\sqrt{2\pi }\sigma }\int_{-\infty }^{\infty }x_{i}^{2}\delta \left(x_{i}\right)p_{x}\left(x_{i}\right)\text{d}x_{i}.$$

The two integrals abbreviated as $I_{i}^{0}$ and $I_{i}^{1}$ are evaluated now. For $I_{i}^{0}$, the substitution *z* = *x_i_/σ* is introduced

(110) $$I_{i}^{0}=\frac{\sigma^{2}}{\sqrt{2\pi }\mu \vartheta }\int_{-\infty }^{\infty }z^{2}{\text{e}}^{-\frac{1}{2}z^{2}}\Phi \left(-\frac{k_{i}\sigma z}{D_{Q}}+{\text{Φ}}^{-1}(\vartheta )\right)\text{d}z.$$

The following integral identity [16] is applied for real parameters *a* and *b*

(111) $$\frac{1}{\sqrt{2\pi }}\int_{-\infty }^{\infty }t^{2}{\text{e}}^{-\frac{1}{2}t^{2}}\text{Φ}(at+b)\text{d}t=\text{Φ}\left(\frac{b}{{\left(1+a^{2}\right)}^{1/2}}\right)-\frac{1}{\sqrt{2\pi }}\frac{a^{2}b}{{\left(1+a^{2}\right)}^{3/2}}{\text{e}}^{-\frac{1}{2}\frac{b^{2}}{1+a^{2}}}.$$

Evaluating (111) with *a* = −*k_i_σ/D_Q_*, *b* = Φ^−1^(ϑ) from (108) yields for the right-hand side of (111)

(112) $$\begin{array}{ll} \text{Φ} & \left(\frac{b}{{\left(1+a^{2}\right)}^{1/2}}\right)-\frac{1}{\sqrt{2\pi }}\frac{a^{2}b}{{\left(1+a^{2}\right)}^{3/2}}{\text{e}}^{-\frac{1}{2}\frac{b^{2}}{1+a^{2}}}\\ & =\text{Φ}\left(\frac{{\text{Φ}}^{-1}(\vartheta )}{{\left(1+{\left(k_{i}\sigma \right)}^{2}/D_{Q}^{2}\right)}^{1/2}}\right)-\frac{1}{\sqrt{2\pi }}\frac{{\left(k_{i}\sigma \right)}^{2}{\text{Φ}}^{-1}(\vartheta )}{D_{Q}^{2}{\left(1+{\left(k_{i}\sigma \right)}^{2}/D_{Q}^{2}\right)}^{3/2}}{\text{e}}^{-\frac{1}{2}\frac{{\left[{\text{Φ}}^{-1}(\vartheta )\right]}^{2}}{1+{\left(k_{i}\sigma \right)}^{2}/D_{Q}^{2}}}. \end{array}$$

Assuming ${\left(k_{i}\sigma \right)}^{2}\ll D_{Q}^{2}$ for large *N* further simplifies (112) and one obtains the result

(113) $$I_{i}^{0}≃\frac{\sigma^{2}}{\mu }\left[1-{\text{Φ}}^{-1}(\vartheta )\left[\frac{{\text{e}}^{-\frac{1}{2}{\left[{\text{Φ}}^{-1}(\vartheta )\right]}^{2}}}{\sqrt{2\pi }\vartheta }\right]\frac{{\left(k_{i}\sigma \right)}^{2}}{D_{Q}^{2}}\right].$$

For (113) the asymptotic generalized progress coefficient definition $e_{\vartheta }^{1,1}$ from (45) can be applied with parameters *a* = 1 and *b* = 1

(114) $$e_{\vartheta }^{1,1}=-{\text{Φ}}^{-1}(\vartheta )\left[\frac{{\text{e}}^{-\frac{1}{2}{\left[{\text{Φ}}^{-1}(\vartheta )\right]}^{2}}}{\sqrt{2\pi }\vartheta }\right].$$

This leads to following result for the first integral $I_{i}^{0}$

(115) $$I_{i}^{0}≃\frac{\sigma^{2}}{\mu }\left[1+e_{\vartheta }^{1,1}\frac{{\left(k_{i}\sigma \right)}^{2}}{D_{Q}^{2}}\right].$$

Second integral $I_{i}^{1}$ from (109) is expressed using expected values over the normal density *p_x_* of the terms given by $x_{i}^{2}\delta (x_{i}).$ With *δ*(*x_i_*) given in Eq. (64) one gets

(116) $$I_{i}^{1}≃-\frac{c_{\vartheta }}{\mu D_{Q}}\left(\text{E}\left[x_{i}^{4}\right]+A\sin\left(\alpha y_{i}\right)\text{E}\left[x_{i}^{2}\sin\left(\alpha x_{i}\right)\right]+A\cos\left(\alpha y_{i}\right)\text{E}\left[x_{i}^{2}\right]-A\cos\left(\alpha y_{i}\right)\text{E}\left[x_{i}^{2}\cos\left(\alpha x_{i}\right)\right]\right).$$

One has $\text{E}\left[x_{i}^{4}\right]=3\sigma^{4}$ and $\text{E}\left[x_{i}^{2}\right]=\sigma^{2}$. Using results from (29) the remaining expected values read

(117) $$\text{E}\left[x_{i}^{2}\sin\left(\alpha x_{i}\right)\right]=0,\;\text{E}\left[x_{i}^{2}\cos\left(\alpha x_{i}\right)\right]=\left(\sigma^{2}-\alpha^{2}\sigma^{4}\right){\text{e}}^{-\frac{1}{2}{\left(\alpha \sigma \right)}^{2}}.$$

Therefore, one gets

(118) $$I_{i}^{1}≃-\frac{c_{\vartheta }\sigma^{2}}{\mu D_{Q}}\left[3\sigma^{2}+A\cos\left(\alpha y_{i}\right)\left(1-{\text{e}}^{-\frac{1}{2}{\left(\alpha \sigma \right)}^{2}}+\alpha^{2}\sigma^{2}{\text{e}}^{-\frac{1}{2}{\left(\alpha \sigma \right)}^{2}}\right)\right].$$

Collecting the results (115) and (118) with *k_i_* = 2*y_i_* and inserting them back into (107) the expected value finally reads

(119) $$\frac{1}{\mu^{2}}E^{\left(2\right)}≃\frac{\sigma^{2}}{\mu }\left\{1+e_{\vartheta }^{1,1}\frac{{\left(2y_{i}\right)}^{2}\sigma^{2}}{D_{Q}^{2}}-\frac{c_{\vartheta }}{D_{Q}}\left[3\sigma^{2}+A\cos\left(\alpha y_{i}\right)\left(1-{\text{e}}^{-\frac{1}{2}{\left(\alpha \sigma \right)}^{2}}+\alpha^{2}\sigma^{2}{\text{e}}^{-\frac{1}{2}{\left(\alpha \sigma \right)}^{2}}\right)\right]\right\}.$$

The solution of the second expected value $\frac{1}{\mu^{2}}E^{\left(1,1\right)}$ from (103) is presented now. First an exact integral is derived. Then, approximations are applied to give closed-form solutions.

### Proposition 3

*Let μ,* λ ∈ ℕ *with μ ≥ 1 and μ* < λ *and let p_x_ denote the PDF of the random mutation x ~* 𝒩 *(0, σ^2^). Let x*_*k*;λ_ *denote the k-th best value (out of* λ*) of the i-th mutation component* (**x**_*k*;λ_)*_i_. Furthermore, let P_Q_ and $P_{Q}^{-1}$ denote the quality gain CDF (and its inverse), respectively, with B denoting the beta function. Then, the second order expected value reads*

(120) $$\begin{array}{ll} \frac{1}{\mu^{2}}\overset{\mu }{\underset{l=2}{\sum }}\overset{l-1}{\underset{k=1}{\sum }} & E\left[x_{k;\text{λ}}x_{l;\text{λ}}\right]=\frac{1}{2}\frac{\text{λ}}{\mu }\frac{\mu -1}{\mu }\int_{-\infty }^{\infty }x_{1}p_{x}\left(x_{1}\right)\int_{-\infty }^{\infty }x_{2}p_{x}\left(x_{2}\right)\\ & \times \left(\frac{1}{\text{B}(\text{λ}-\mu ,\mu )}\int_{0}^{1}t^{\text{λ}-\mu -1}{\left(1-t\right)}^{\mu -2}P_{Q}\left(P_{Q}^{-1}(1-t)|x_{1}\right)P_{Q}\left(P_{Q}^{-1}(1-t)|x_{2}\right)\text{d}t\right)\text{d}x_{2}\text{d}x_{1} \end{array}$$

### Proof

First, a joint order statistic density has to be derived for the expected value. Then, the double sum is converted into a single integral using a known identity. The resulting five-fold integration is restructured by exchanging bounds and then successively solved.

Starting with (103), the double sum includes mixed contributions from the *k*-th and *l*-th best elements of the *i*-th mutation component. To avoid confusion with the summation indices *k* and *l*, the integration variables associated with *k*-th element will be denoted as *x*_1_ (mutation) and *q*_1_ (quality), while the *l*-th element is integrated over *x*_2_ and *q*_2_. The ordering 1 ≤ *k* < *l* ≤ *λ* is assumed with *k* yielding a smaller (better) quality value *q*_1_ < *q*_2_. Additionally, the joint probability density *p_k,l;λ_*(*x*_1_, *x*_2_) is needed, such that the expected value can be formulated as

(121) $$\frac{1}{\mu^{2}}E^{\left(1,1\right)}=\frac{1}{\mu^{2}}\overset{\mu }{\underset{l=2}{\sum }}\overset{l-1}{\underset{k=1}{\sum }}\int_{-\infty }^{\infty }\int_{-\infty }^{\infty }x_{1}x_{2}p_{k,l;\text{λ}}\left(x_{1},x_{2}\right)\text{d}x_{2}\text{d}x_{1}.$$

The mutation densities are independent and denoted by *p_x_*(*x*_1_) and *p_x_*(*x*_2_), respectively. Given mutation components *x*_1_ and *x*_2_, the conditional density obtaining the quality values *q*_1_ and *q*_2_ is *p_Q_* (*q*_1_|*x*_1_) and *p_Q_* (*q*_2_|*x*_2_), respectively. Given *q*_1_ and *q*_2_, one has *k* − 1 values smaller than *q*_1_, *l* − *k* − 1 values between *q*_1_ and *q*_2_ and λ − *l* values larger than *q*_2_ with probabilities

(122) $$\begin{array}{lll} \mathrm{Pr}{\left\{Q\leq q_{1}\right\}}^{k-1} & = & P_{Q}{\left(q_{1}\right)}^{k-1}\\ \mathrm{Pr}{\left\{q_{1}\leq Q\leq q_{2}\right\}}^{l-k-1} & = & {\left[P_{Q}\left(q_{2}\right)-P_{Q}\left(q_{1}\right)\right]}^{l-k-1}\\ \mathrm{Pr}{\left\{Q>q_{2}\right\}}^{\text{λ}-1} & = & {\left[1-P_{Q}\left(q_{2}\right)\right]}^{\text{λ}-l}\text{, } \end{array}$$

and *P_Q_* (*q*) denoting the quality gain CDF. The joint probability density can therefore be written as

(123) $$\begin{array}{lll} p_{k,l;\text{λ}}\left(x_{1},x_{2}\right) & = & p_{x}\left(x_{1}\right)p_{x}\left(x_{2}\right)\int_{q_{\min}}^{\infty }p_{Q}\left(q_{1}|x_{1}\right)\int_{q_{1}}^{\infty }p_{Q}\left(q_{2}|x_{2}\right)\\ & & \times \text{λ}!\frac{P_{Q}{\left(q_{1}\right)}^{k-1}{\left[P_{Q}\left(q_{2}\right)-P_{Q}\left(q_{1}\right)\right]}^{l-k-1}{\left[1-P_{Q}\left(q_{2}\right)\right]}^{\text{λ}-l}}{(k-1)!(l-k-1)!(\text{λ}-l)!}\text{d}q_{2}\text{d}q_{1}, \end{array}$$

with integration ranges *q*_min_ ≤ *q*_1_ < ∞ and *q*_1_ < *q*_2_ < ∞ as *k* < *l*. Lower bound *q*_min_ denotes the smallest possible quality value, which is resolved later. The factorials exclude the irrelevant combinations among the three groups given in (122). Plugging (123) into (121) and moving the sum into the innermost integral gives

(124) $$\begin{array}{ll} \frac{1}{\mu^{2}}E^{\left(1,1\right)}=\frac{\text{λ}!}{\mu^{2}} & \int_{-\infty }^{\infty }x_{1}p_{x}\left(x_{1}\right)\int_{-\infty }^{\infty }x_{2}p_{x}\left(x_{2}\right)\int_{q_{\min}}^{\infty }p_{Q}\left(q_{1}|x_{1}\right)\int_{q_{1}}^{\infty }p_{Q}\left(q_{2}|x_{2}\right)\\ & \times \sum_{l=2}^{\mu }\sum_{k=1}^{l-1}\frac{P_{Q}{\left(q_{1}\right)}^{k-1}{\left[P_{Q}\left(q_{2}\right)-P_{Q}\left(q_{1}\right)\right]}^{l-k-1}{\left[1-P_{Q}\left(q_{2}\right)\right]}^{\text{λ}-l}}{(k-1)!(l-k-1)!(\text{λ}-l)!}\text{d}q_{2}\;\text{d}q_{1}\;\text{d}x_{2}\;\text{d}x_{1}. \end{array}$$

The double sum of (124) over the *P_Q_* -values will be expressed by an integral. This can be done using an identity from [4, p. 113]. Setting *ν* = 2 and identifying the indices as *i*_1_ = *l* and *i*_2_ = *k*, the identity yields

(125) $$\overset{\mu }{\underset{l=2}{\sum }}\overset{l-1}{\underset{k=1}{\sum }}\frac{Q_{1}^{\text{λ}-l}{\left[Q_{2}-Q_{1}\right]}^{l-k-1}{\left[1-Q_{2}\right]}^{k-1}}{(\text{λ}-l)!(l-k-1)!(k-1)!}=\frac{1}{(\text{λ}-\mu -1)!(\mu -2)!}\int_{0}^{Q_{1}}t^{\text{λ}-\mu -1}{\left(1-t\right)}^{\mu -2}\text{d}t,$$

for real values *Q*_1_ and *Q*_2_, with integers *ν* ≤ *μ* < λ. Now the substitution *Q*_1_ = 1 − *P_Q_* (*q*_2_), *Q*_2_ = 1 − *P_Q_* (*q*_1_) can be performed and the double sum of (124) can be recognized by comparing with (125). Applying the identity therefore yields

(126) $$\overset{\mu }{\underset{l=2}{\sum }}\overset{l-1}{\underset{k=1}{\sum }}\frac{{\left[1-P_{Q}\left(q_{2}\right)\right]}^{\text{λ}-l}{\left[P_{Q}\left(q_{2}\right)-P_{Q}\left(q_{1}\right)\right]}^{l-k-1}{\left[P_{Q}\left(q_{1}\right)\right]}^{k-1}}{(\text{λ}-l)!(l-k-1)!(k-1)!}=\frac{1}{(\text{λ}-\mu -1)!(\mu -2)!}\int_{0}^{1-P_{Q}\left(q_{2}\right)}t^{\text{λ}-\mu -1}(1-t{)}^{\mu -2}\text{d}t.$$

Hence, Eq. (124) is expressed as

(127) $$\begin{array}{lll} \frac{1}{\mu^{2}}E^{\left(1,1\right)} & = & \frac{\text{λ}!}{\mu^{2}}\frac{1}{(\text{λ}-\mu -1)!(\mu -2)!}\int_{-\infty }^{\infty }x_{1}p_{x}\left(x_{1}\right)\int_{-\infty }^{\infty }x_{2}p_{x}\left(x_{2}\right)\\ & & \times \int_{q_{\min}}^{\infty }p_{Q}\left(q_{1}|x_{1}\right)\int_{q_{1}}^{\infty }p_{Q}\left(q_{2}|x_{2}\right)\int_{0}^{1-P_{Q}\left(q_{2}\right)}t^{\text{λ}-\mu -1}{\left(1-t\right)}^{\mu -2}\text{d}t\;\text{d}q_{2}\;\text{d}q_{1}\;\text{d}x_{2}\;\text{d}x_{1}. \end{array}$$

The prefactor of Eq. (127) can be evaluated as

(128) $$\frac{\text{λ}!}{\mu^{2}}\frac{1}{(\text{λ}-\mu -1)!(\mu -2)!}=\frac{\text{λ}(\text{λ}-1)!(\mu -1)}{\mu^{2}(\text{λ}-\mu -1)!(\mu -1)!}=\frac{1}{\vartheta }\frac{\mu -1}{\mu }\frac{1}{\text{B}(\text{λ}-\mu ,\mu )}.$$

Now the integration order will be exchanged twice in (127). First the order between *t* and *q*_2_ is exchanged. Then the order between *t* and *q*_1_ is exchanged, such that both *q*-integrations are performed before the *t*-integration enabling the application of the large population identity (37). Starting with integration bounds

(129) $$q_{1}\leq q_{2}<\infty ,\;0\leq t\leq 1-P_{Q}\left(q_{2}\right),$$

and using the inverse function $P_{Q}^{-1}$ with $q_{2}=P_{Q}^{-1}(1-t)$ the exchanged bounds between *t* and *q*_2_ are

(130) $$0\leq t\leq 1-P_{Q}\left(q_{1}\right),\;q_{1}\leq q_{2}\leq P_{Q}^{-1}(1-t).$$

Using factor (128) and exchanged bounds (130), the expression (127) is reformulated as

(131) $$\begin{array}{lll} \frac{1}{\mu^{2}}E^{\left(1,1\right)} & = & \frac{1}{\vartheta }\frac{\mu -1}{\mu }\frac{1}{\text{B}(\text{λ}-\mu ,\mu )}\int_{-\infty }^{\infty }x_{1}p_{x}\left(x_{1}\right)\int_{-\infty }^{\infty }x_{2}p_{x}\left(x_{2}\right)\\ & & \times \int_{q_{\min}}^{\infty }p_{Q}\left(q_{1}|x_{1}\right)\int_{0}^{1-P_{Q}\left(q_{1}\right)}t^{\text{λ}-\mu -1}{\left(1-t\right)}^{\mu -2}\int_{q_{1}}^{P_{Q}^{-1}(1-t)}p_{Q}\left(q_{2}|x_{2}\right)\text{d}q_{2}\;\text{d}t\;\text{d}q_{1}\;\text{d}x_{2}\;\text{d}x_{1}. \end{array}$$

Now the integration order between *t* and *q*_1_ is exchanged starting from

(132) $$q_{\min}\leq q_{1}<\infty ,\;0\leq t\leq 1-P_{Q}\left(q_{1}\right),$$

yielding exchanged bounds

(133) $$0\leq t\leq 1,\;q_{\min}\leq q_{1}\leq P_{Q}^{-1}(1-t).$$

Therefore, one arrives at the following integral to be solved (beta function has been moved inside as it will be evaluated during the *t*-integration)

(134) $$\begin{array}{lll} \frac{1}{\mu^{2}}E^{\left(1,1\right)} & = & \frac{1}{\vartheta }\frac{\mu -1}{\mu }\int_{-\infty }^{\infty }x_{1}p_{x}\left(x_{1}\right)\int_{-\infty }^{\infty }x_{2}p_{x}\left(x_{2}\right)\\ & & \times (\frac{1}{\text{B}(\text{λ}-\mu ,\mu )}\int_{0}^{1}t^{\text{λ}-\mu -1}{\left(1-t\right)}^{\mu -2}\\ & & \times \left[\int_{q_{\min}}^{P_{Q}^{-1}(1-t)}p_{Q}\left(q_{1}|x_{1}\right)\left\{\int_{q_{1}}^{P_{Q}^{-1}(1-t)}p_{Q}\left(q_{2}|x_{2}\right)\text{d}q_{2}\right\}\text{d}q_{1}\right]\text{d}t)\text{d}x_{2}\;\text{d}x_{1}. \end{array}$$

Now the integrals in (134) will be successively solved. Starting with integral {·} over *q*_2_ one has

(135) $$\int_{q_{1}}^{P_{Q}^{-1}(1-t)}p_{Q}\left(q_{2}|x_{2}\right)\text{d}q_{2}={\left[P_{Q}\left(q_{2}|x_{2}\right)\right]}_{q_{1}}^{P_{Q}^{-1}(1-t)}=P_{Q}\left(P_{Q}^{-1}(1-t)|x_{2}\right)-P_{Q}\left(q_{1}|x_{2}\right).$$

The *q*_1_-integration within [·] using (135) yields

(136) $$\int_{q_{\min}}^{P_{Q}^{-1}(1-t)}p_{Q}\left(q_{1}|x_{1}\right)\left(P_{Q}\left(P_{Q}^{-1}(1-t)|x_{2}\right)-P_{Q}\left(q_{1}|x_{2}\right)\right)\text{d}q_{1}$$

(137) $$=P_{Q}\left(P_{Q}^{-1}(1-t)|x_{2}\right)\int_{q_{\min}}^{P_{Q}^{-1}(1-t)}p_{Q}\left(q_{1}|x_{1}\right)\text{d}q_{1}$$

(138) $$-\int_{q_{\min}}^{P_{Q}^{-1}(1-t)}p_{Q}\left(q_{1}|x_{1}\right)P_{Q}\left(q_{1}|x_{2}\right)\text{d}q_{1}.$$

First integral (137) is easily evaluated, as the conditional density is integrated over its support giving

(139) $$\begin{array}{lll} P_{Q}\left(P_{Q}^{-1}(1-t)|x_{2}\right)\int_{q_{\min}}^{P_{Q}^{-1}(1-t)}p_{Q}\left(q_{1}|x_{1}\right)\text{d}q_{1} & = & P_{Q}\left(P_{Q}^{-1}(1-t)|x_{2}\right){\left[P_{Q}\left(q_{1}|x_{1}\right)\right]}_{q_{\min}}^{P_{Q}^{-1}(1-t)}\\ & = & P_{Q}\left(P_{Q}^{-1}(1-t)|x_{2}\right)P_{Q}\left(P_{Q}^{-1}(1-t)|x_{1}\right), \end{array}$$

with *P_Q_* (*q*_min_|*x*_1_) = Pr{*Q* ≤ *q*_min_|*x*_1_} = 0. Note that the resulting factors are equal up to the conditional variables *x*_1_ and *x*_2_.

The second integral (138) will be simplified using integration by parts. Thereafter, one can exchange the *x*_1_ and *x*_2_ variables to find a simpler expression for the original integral. Integration by parts yields

(140) $$\int_{q_{\min}}^{P_{Q}^{-1}(1-t)}p_{Q}\left(q_{1}|x_{1}\right)P_{Q}\left(q_{1}|x_{2}\right)\text{d}q_{1}=P_{Q}\left(P_{Q}^{-1}(1-t)|x_{1}\right)P_{Q}\left(P_{Q}^{-1}(1-t)|x_{2}\right)-\int_{q_{\min}}^{P_{Q}^{-1}(1-t)}P_{Q}\left(q_{1}|x_{1}\right)p_{Q}\left(q_{1}|x_{2}\right)\text{d}q_{1}.$$

Equation (140) inserted into (134) has to be integrated over *x*_1_ and *x*_2_, of which the order can be exchanged. For the following step the *t*-integration and the prefactors of (134) have no influence, such that they are dropped for better readability. Integrating both sides of (140) yields

(141) $$\begin{array}{l} \int_{-\infty }^{\infty }x_{1}p_{x}\left(x_{1}\right)\int_{-\infty }^{\infty }x_{2}p_{x}\left(x_{2}\right)\int_{q_{\min}}^{P_{Q}^{-1}(1-t)}p_{Q}\left(q_{1}|x_{1}\right)P_{Q}\left(q_{1}|x_{2}\right)\text{d}q_{1}\text{d}x_{2}\text{d}x_{1}\\ =\int_{-\infty }^{\infty }x_{1}p_{x}\left(x_{1}\right)\int_{-\infty }^{\infty }x_{2}p_{x}\left(x_{2}\right)P_{Q}\left(P_{Q}^{-1}(1-t)|x_{1}\right)P_{Q}\left(P_{Q}^{-1}(1-t)|x_{2}\right)\text{d}x_{2}\text{d}x_{1}\\ -\int_{-\infty }^{\infty }x_{2}p_{x}\left(x_{2}\right)\int_{-\infty }^{\infty }x_{1}p_{x}\left(x_{1}\right)\int_{q_{\min}}^{P_{Q}^{-1}(1-t)}P_{Q}\left(q_{1}|x_{2}\right)p_{Q}\left(q_{1}|x_{1}\right)\text{d}q_{1}\text{d}x_{1}\text{d}x_{2}, \end{array}$$

where in the last line the integration order of *x*_1_ and *x*_2_ was exchanged, such that an expression equivalent to the left-hand side of (141) is obtained with given arguments for *p_Q_* and *P_Q_*. Collecting the terms, Eq. (141) can be formulated as

(142) $$\int_{-\infty }^{\infty }x_{1}p_{x}\left(x_{1}\right)\int_{-\infty }^{\infty }x_{2}p_{x}\left(x_{2}\right)\int_{q_{\min}}^{P_{Q}^{-1}(1-t)}p_{Q}\left(q_{1}|x_{1}\right)P_{Q}\left(q_{1}|x_{2}\right)\text{d}q_{1}\;\text{d}x_{2}\;\text{d}x_{1}=\frac{1}{2}\int_{-\infty }^{\infty }x_{1}p_{x}\left(x_{1}\right)\int_{-\infty }^{\infty }x_{2}p_{x}\left(x_{2}\right)P_{Q}\left(P_{Q}^{-1}(1-t)|x_{1}\right)P_{Q}\left(P_{Q}^{-1}(1-t)|x_{2}\right)\text{d}x_{2}\;\text{d}x_{1}.$$

Noting that the right-hand side of result (142) is one half of the first integration result (139) after *x*-integration and noting the minus sign in (138), one gets for (136) the expression

(143) $$\begin{array}{l} \int_{-\infty }^{\infty }x_{1}p_{x}\left(x_{1}\right)\int_{-\infty }^{\infty }x_{2}p_{x}\left(x_{2}\right)\int_{q_{\min}}^{P_{Q}^{-1}(1-t)}p_{Q}\left(q_{1}|x_{1}\right)\left(P_{Q}\left(P_{Q}^{-1}(1-t)|x_{2}\right)-P_{Q}\left(q_{1}|x_{2}\right)\right)\text{d}q_{1}\;\text{d}x_{2}\;\text{d}x_{1}\\ =\int_{-\infty }^{\infty }x_{1}p_{x}\left(x_{1}\right)\int_{-\infty }^{\infty }x_{2}p_{x}\left(x_{2}\right)\left(1-\frac{1}{2}\right)P_{Q}\left(P_{Q}^{-1}(1-t)|x_{1}\right)P_{Q}\left(P_{Q}^{-1}(1-t)|x_{2}\right)\text{d}x_{2}\;\text{d}x_{1}. \end{array}$$

Inserting the results of (143) back into [·] of (134) and including all prefactors, the five-fold integral simplifies providing the desired result of Eq. (120).

### Theorem 4

*Let p_x_ denote the density of the i-th component mutation x* ~ 𝒩 (0, *σ*^2^) *and let x*_*k*;λ_ *denote the k-th best value (out of* λ*) of the i-th mutation component* (**x**_*k*;*λ*_)_*i*_. *Let P_Q_ denote the quality gain CDF with its quantile function given by $P_{Q}^{-1}.$ For a truncation ratio ϑ the limit of the second order expected value reads*

(144) $$\underset{\begin{array}{c} \mu ,\text{λ}\to \infty \\ \vartheta =\text{const}. \end{array}}{\lim}\frac{1}{\mu (\mu -1)}\sum_{l=2}^{\mu }\;\sum_{k=1}^{l-1}\text{E}\left[x_{k;\text{λ}}x_{l;\text{λ}}\right]=\frac{1}{2}{\left[\frac{1}{\vartheta }\int_{-\infty }^{\infty }x_{i}p_{x}\left(x_{i}\right)P_{Q}\left(P_{Q}^{-1}(\vartheta )|x_{i}\right)\text{d}x_{i}\right]}^{2}.$$

### Proof

Starting from Eq. (120) the *μ*-dependent prefactor was rearranged in a way that the factor (*μ* − 1)/*μ* in (120) is retained in the final result. Formally one could include (*μ* − 1)/*μ* in the sequence (38) and take the limit. However, it is desirable to keep the factor in the progress rate as a correction for finite *μ*-values. As a next step, one can define $f_{x}(t)=P_{Q}(P_{Q}^{-1}(1-t)|x_{1})P_{Q}(P_{Q}^{-1}(1-t)|x_{2}).$ As 0 ≤ *f_x_*(*t*) ≤ 1 the same bound estimation as in (43) holds. Furthermore, both mutation integrals over density *p_x_* are finite, see also (44). Therefore, the limit is evaluated with $f_{x}(t){|}_{t=1-\vartheta }=P_{Q}(P_{Q}^{-1}(\vartheta )|x_{1})P_{Q}(P_{Q}^{-1}(\vartheta )|x_{2})$ and *a* = 2 as

(145) $$\begin{array}{lll} \underset{\begin{array}{c} \mu ,\text{λ}\to \infty \\ \vartheta =\text{ const}. \end{array}}{\lim}\frac{1}{\mu (\mu -1)}E^{\left(1,1\right)} & = & \frac{1}{2}\frac{1}{\vartheta^{2}}\int_{-\infty }^{\infty }x_{1}p_{x}\left(x_{1}\right)\int_{-\infty }^{\infty }x_{2}p_{x}\left(x_{2}\right)P_{Q}\left(P_{Q}^{-1}(\vartheta )|x_{1}\right)P_{Q}\left(P_{Q}^{-1}(\vartheta )|x_{2}\right)\text{d}x_{2}\text{d}x_{1}\\ & = & \frac{1}{2}\frac{1}{\vartheta^{2}}\int_{-\infty }^{\infty }x_{1}p_{x}\left(x_{1}\right)P_{Q}\left(P_{Q}^{-1}(\vartheta )|x_{1}\right)\text{d}x_{1}\int_{-\infty }^{\infty }x_{2}p_{x}\left(x_{2}\right)P_{Q}\left(P_{Q}^{-1}(\vartheta )|x_{2}\right)\text{d}x_{2}\\ & = & \frac{1}{2}{\left[\frac{1}{\vartheta }\int_{-\infty }^{\infty }x_{i}p_{x}\left(x_{i}\right)P_{Q}\left(P_{Q}^{-1}(\vartheta )|x_{i}\right)\text{d}x_{i}\right]}^{2}, \end{array}$$

with *x_i_* re-introduced in the last line to denote the *i*-th mutation component, which gives Eq. (144).

In [·] of result (144), one can identify the first order progress rate −*φ_i_* within the large population limit derived in Eq. (60). Refactoring (144) to obtain $\frac{1}{\mu^{2}}E^{\left(1,1\right)},$ one can insert the *φ_i_*-approximation from (96). Noting that $c_{\vartheta }^{2}=e_{\vartheta }^{2,0}$ via (45), one gets

(146) $$\begin{array}{lll} \frac{1}{\mu^{2}}E^{\left(1,1\right)} & ≃ & \frac{1}{2}\frac{\mu -1}{\mu }\varphi_{i}^{2}\\ & ≃ & \frac{1}{2}\frac{\mu -1}{\mu }e_{\vartheta }^{2,0}\frac{\sigma^{4}}{D_{Q}^{2}}{\left(2y_{i}+{\text{e}}^{-\frac{1}{2}{\left(\alpha \sigma \right)}^{2}}\alpha \;A\;\sin\left(\alpha \;y_{i}\right)\right)}^{2}. \end{array}$$

Finally, inserting the results from (119) and (146) into (101), one obtains the second order progress rate

(147) $$\begin{array}{l} \varphi_{i}^{\text{II}}≃c_{\vartheta }\frac{\sigma^{2}}{D_{Q}}\left(4y_{i}^{2}+{\text{e}}^{-\frac{1}{2}{\left(\alpha \sigma \right)}^{2}}2\alpha \;Ay_{i}\;\sin\left(\alpha \;y_{i}\right)\right)\\ \;-\frac{\sigma^{2}}{\mu }\{1+e_{\vartheta }^{1,1}\frac{{\left(2y_{i}\right)}^{2}\sigma^{2}}{D_{Q}^{2}}-\frac{c_{\vartheta }}{D_{Q}}\left[3\sigma^{2}+A\cos\left(\alpha \;y_{i}\right)\left(1-{\text{e}}^{-\frac{1}{2}{\left(\alpha \sigma \right)}^{2}}+\alpha^{2}\sigma^{2}{\text{e}}^{-\frac{1}{2}{\left(\alpha \sigma \right)}^{2}}\right)\right]\\ \;+(\mu -1)e_{\vartheta }^{2,0}\frac{\sigma^{2}}{D_{Q}^{2}}{\left(2y_{i}+{\text{e}}^{-\frac{1}{2}{\left(\alpha \sigma \right)}^{2}}\alpha A\sin\left(\alpha \;y_{i}\right)\right)}^{2}\}, \end{array}$$

which serves as an approximation in the asymptotic limit of infinitely large dimensionality and population size. However, experimental investigations will also show good agreement for finite *N*, *μ*, and λ.

For future investigations of the convergence and step-size adaptation properties of the (*μ*/*μ_I_*,λ)-ES, a simpler expression than (147) is needed. To this end, the *N*-dependency of the terms within {·} of (147) is investigated. It will be shown that for *N* → ∞ and *μ* = *o* (*N*) only the term −*σ*^2^*/μ* yields relevant contributions. The relevant terms in {·} of Eq. (147) are abbreviated according to their respective factors as $e_{\vartheta }^{1,1},c_{\vartheta }/D_{Q}$, *c_ϑ_*/*D_Q_* and $e_{\vartheta }^{2,0}$. In order to maximize the absolute value of the individual terms a lower bound for $D_{Q}^{2}$ is needed. Given the form of $D_{Q}^{2}$ from Eq. (31), no useful lower bound for the variance could be established satisfying $D_{Q}^{2}$ > 0 for any *y_i_* due to the trigonometric terms. Therefore, we will restrict the analysis to the sphere limit case *A* → 0. This assumption might seem crude. However, the most important characteristics are already contained in the first *φ_i_*-dependent term of (147) referred to as the *gain* term in sphere model theory [5]. On the other hand, the *loss* terms in {·} are mostly dominated by the first term −*σ*^2^*/μ*. Experiments will affirm this assumption.

As the $\varphi_{i}^{\text{II}}$-approximation shall be valid for a constant σ* given any *R*-value, the mutation strength is re-normalized using (4)

(148) $$\sigma =\frac{\sigma^{*}R}{N}.$$

Setting *A* = 0, *σ* = σ*R/N, and $\sum_{i}y_{i}^{2}=R^{2}$ in (31), one obtains the sphere variance for constant normalized mutation strength as

(149) $$\begin{array}{lll} D_{Q,\text{ sph }}^{2} & = & \overset{N}{\underset{i=1}{\sum }}\left[4\sigma^{2}y_{i}^{2}+2\sigma^{4}\right]=4\sigma^{2}R^{2}+2N\sigma^{4}=4R^{4}{\left(\frac{\sigma^{*}}{N}\right)}^{2}+2N{\left(\frac{\sigma^{*}R}{N}\right)}^{4}\\ & = & 4R^{4}{\left(\frac{\sigma^{*}}{N}\right)}^{2}\left(1+\frac{\sigma^{*2}}{2N}\right). \end{array}$$

In the limit *N* → ∞ the second term of (149) is negligible for constant σ* giving

(150) $$D_{Q,\text{sph}}^{2}≃4R^{4}{\left(\frac{\sigma^{*}}{N}\right)}^{2}.$$

Having obtained the sphere variance asymptotic in (150), the terms within {·} of (147) are evaluated. The term with prefactor $e_{\vartheta }^{1,1}$ yields with *σ* = σ*R/*N* and using (150)

(151) $$e_{\vartheta }^{1,1}\frac{\sigma^{2}}{D_{Q}^{2}}{\left(2y_{i}\right)}^{2}=e_{\vartheta }^{1,1}\frac{{\left(\sigma^{*}\frac{R}{N}\right)}^{2}}{4R^{4}{\left(\sigma^{*}/N\right)}^{2}}{\left(2y_{i}\right)}^{2}=e_{\vartheta }^{1,1}\frac{y_{i}^{2}}{R^{2}}=O\left(\frac{1}{N}\right).$$

It was used in (151) that a single component $y_{i}^{2}$ contributes in expectation 1/*N* to the residual distance $R^{2}=\sum_{j=1}^{N}y_{j}^{2},$ see also (12). The second term with prefactor *c_ϑ_*/*D_Q_* using *D_Q_* ≃ 2*R*^2^σ*/N with *A* = 0 as

(152) $$\frac{3c_{\vartheta }{\left(\sigma^{*}\frac{R}{N}\right)}^{2}}{D_{Q}}=\frac{3c_{\vartheta }{\left(\sigma^{*}\frac{R}{N}\right)}^{2}}{2R^{2}\sigma^{*}/N}=O\left(\frac{1}{N}\right).$$

The last term with prefactor $e_{\vartheta }^{2,0}$ yields with *A* = 0 and using (150)

(153) $$\begin{array}{lll} (\mu -1)e_{\vartheta }^{2,0}\frac{\sigma^{2}}{D_{Q}^{2}}{\left(2y_{i}\right)}^{2} & = & (\mu -1)e_{\vartheta }^{2,0}\frac{{\left(\sigma^{*}\frac{R}{N}\right)}^{2}}{4R^{4}{\left(\sigma^{*}/N\right)}^{2}}{\left(2y_{i}\right)}^{2}\\ & = & (\mu -1)e_{\vartheta }^{2,0}\frac{y_{i}^{2}}{R^{2}}=\{\begin{array}{l} O\left(\frac{1}{N}\right)\;\text{if}\;\mu (N)=\text{const}.\\ O\left(\frac{\mu (N)}{N}\right)\;\text{else}. \end{array} \end{array}$$

In (153) the notation *μ*(*N*) was introduced to emphasize that the population size is usually chosen depending on the dimensionality of the search space. Finally, inserting the results of the loss term investigation for the three terms (151), (152), and (153) back into progress rate (147), one gets for the loss term in {·} of (147)

(154) $$-\frac{\sigma^{2}}{\mu }\left\{1+O\left(\frac{1}{N}\right)+O\left(\frac{\mu (N)}{N}\right)\right\}.$$

Provided that the population size *μ* = *o* (*N*), i.e., increasing sub-linearly with *N*, all terms except “1” in {·} can be neglected for *N* → ∞. Theoretical results concerning population sizing, i.e., choosing the necessary *μ*(*N*) to achieve high global convergence probability (success probability), are not available at this point. It is one of the main future goals of the current research project. Note that treating *μ* as a constant is also not satisfactory, since for large *N* an increase of *μ* is necessary to maintain a high success rate on a highly multimodal problem. However, experimental investigations on the Rastrigin function including step-size adaptation suggest a sub-linear relation, which validates the approximation. Finally, the lengthy result (147) is simplified using the loss term asymptotic of (154) and the second order progress rate approximation is obtained.

#### Second order progress rate

*The second order component-wise progress rate on the Rastrigin function in the asymptotic limits of infinitely large population size μ (constant ϑ = μ*/λ*) and infinitely large dimensionality N with μ =* *o* (*N*) *yields*

(155) $$\varphi_{i}^{\text{II}}≃2y_{i}\varphi_{i}-\frac{\sigma^{2}}{\mu }$$

(156) $$≃c_{\vartheta }\frac{\sigma^{2}}{D_{Q}}\left(4y_{i}^{2}+{\text{e}}^{-\frac{1}{2}{\left(\alpha \sigma \right)}^{2}}2\alpha \;A\;y_{i}\;\sin\;\left(\alpha \;y_{i}\right)\right)-\frac{\sigma^{2}}{\mu }.$$

The expressions for $c_{\vartheta }=e_{\vartheta }^{1,0}$ from (45) and *D_Q_* from (31) were not inserted to improve readability. The first line (155) emphasizes the dependence of $\varphi_{i}^{\text{II}}(\varphi_{i})$ and can be thought of as a more general formula provided that *φ_i_* is known and the loss term behaves similarly to the sphere function loss term −*σ*^2^*/μ*. The second line (156) shows the explicit results for the Rastrigin function. The results (155) and (156) can be mapped to the Evolutionary Progress Principle [5] as the expressions contain a progress gain and loss term, respectively. Here, the gain part scales with *c_ϑ_* and it is a *y_i_*-dependent expression. Hence, depending on the sign of *y_i_* sin (*α y_i_*) it may also yield negative contributions due to local attraction moving the ES away from the global optimizer, cf. Fig. 3. The loss term −*σ*^2^/*μ* is characteristic for intermediate recombination. It introduces significant loss for large *σ*, but can be decreased using a larger *μ* due to recombination effects.

**Fig. 6:**
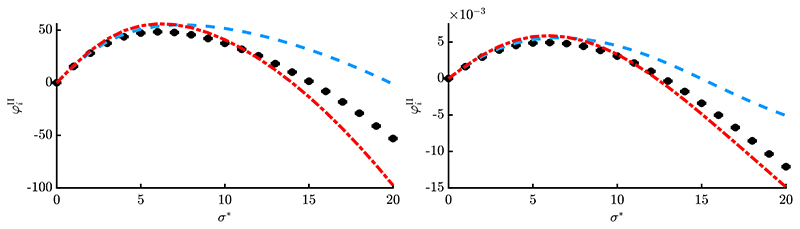
Second order progress rate $\varphi_{i}^{\text{II}}$ as a function of σ* for (10/10,40)-ES with *N* = 20, *A* = 1, *α* = 2*π*, at two residual distances $R=10\sqrt{N}$ with *y_i_* = 11.6 (left) and $R=0.1\sqrt{N}$ with *y_i_* = 0.116 (right). The dashed blue curves show Eq. (147) and the dash-dotted red curves Eq. (156).

**Fig. 7:**
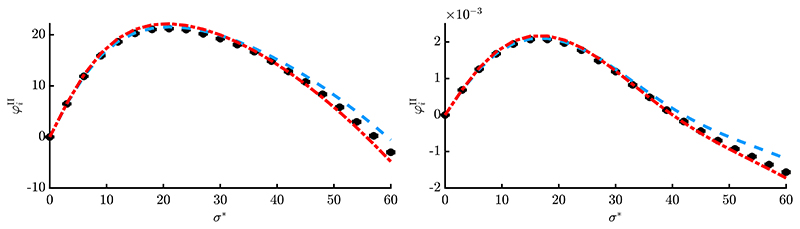
Second order progress rate $\varphi_{i}^{\text{II}}$ as a function of σ* for (100/100, 200)-ES with *N* = 100, *A* = 1, *α* = 2*π*, at two residual distances $R=10\sqrt{N}$ with *y_i_* = 11.9 (left) and $R=0.1\sqrt{N}$ with *y_i_* = 0.119 (right). The dashed blue curves show Eq. (147) and the dash-dotted red curves Eq. (156).

Results of one-generation experiments are presented in Figs. 6 and 7 by evaluating (8) over 10^6^ trials (black dots with vanishing error bars) and comparing with the obtained approximations. The red dash-dotted line is showing simplified result (156), while the blue dashed line is showing (147). The positions **y** were initialized randomly (given *R*) and kept constant over all repetitions. Fig. 6 shows a smaller dimensionality *N* = 20 and truncation ratio *ϑ* = 1/4, while Fig. 7 shows larger values *N* = 100 with *ϑ* = 1/2. This was done to exemplarily investigate the results at different parameter sets.

First thing to note is that the loss term allows negative progress for large σ*, which was not the case for *φ_i_*. The approximation quality is good for different *R*-values (see left and right plots, respectively) and improves for larger *N* and *μ* in Fig. 7, which was expected. Simplified expression $\varphi_{i}^{\text{II}}$ from (156) [red, dash-dotted] yields good results compared to (147) [blue, dashed], with (147) giving slightly better results for smaller σ* and (156) better results at larger σ*. This indicates that additional terms of the Taylor expansion (70) would be needed to further improve the results of (147). However, this would make the expression more involved, which is not desired. Furthermore, the results of Fig. 6 are relatively good considering that a rather small population (10/10, 40)-ES was used at low dimensionality *N* = 20. One can conclude that (156) yields very good results considering its “simplicity”. It will therefore be used in Sec. 5 to investigate the dynamical behavior of the ES. It should be noted that at this point there is no aggregated progress measure over all *N* components, such as the *R*-dependent sphere progress rate. Given some **y**^(*g*)^ one can evaluate all *i* = 1, …, *N* values for $\varphi_{i}^{\text{II}}$ and obtain a progress vector, but the overall effect on *R*^(*g*)^ → *R*^(*g*+1)^ is not known. This will be part of future research. However, the cumulative effect of all *N* progress rates can be evaluated within a dynamical systems model to be shown in the next chapter.

## 5 Evolution equations

In the previous sections one-generation experiments were conducted and compared against progress rate results (96), (147), and (156). In order to have an aggregated measure over all components and many generations, *φ_i_* and $\varphi_{i}^{\text{II }}$ will be used within the evolution equations and compared to real optimization runs of Algorithm 1. Using this method the (mean) global convergence behavior can be investigated.

Given definitions for first and second order progress (7) and (8), the expressions can be reformulated as stochastic iterative mappings between two generations *g* → *g* + 1 according to

(157) $$y_{i}^{\left(g+1\right)}=y_{i}^{\left(g\right)}-\varphi_{i}\left(\sigma^{\left(g\right)},{\text{y}}^{\left(g\right)}\right)+\epsilon^{\left(1\right)}\left(\sigma^{\left(g\right)},{\text{y}}^{\left(g\right)}\right)$$

(158) $${\left(y_{i}^{\left(g+1\right)}\right)}^{2}={\left(y_{i}^{\left(g\right)}\right)}^{2}-\varphi_{i}^{\text{II}}\left(\sigma^{\left(g\right)},{\text{y}}^{\left(g\right)}\right)+\epsilon^{\left(2\right)}\left(\sigma^{\left(g\right)},{\text{y}}^{\left(g\right)}\right).$$

The two terms *ϵ*^(1)^ and *ϵ*^(2)^ can be interpreted as fluctuations w.r.t. the expected values (provided by *φ_i_* and $\varphi_{i}^{\text{II}}$). Thus, it holds E[*ϵ*^(1)^] = 0 = E[*ϵ*^(2)^]. However, the exact transition densities for *g* → *g* + 1 are not known at this point. In principle, they could be approximated using a finite number of higher order moments (or cumulants) to model the fluctuations [5, Ch. 7]. However, for a first study of the progress rate results on the dynamics, the fluctuations are neglected by setting *ϵ*^(1)^ = 0 = *ϵ*^(2)^. Therefore, one arrives at the (deterministic) equations describing the mean-value dynamics of the parental position coordinates

(159) $$y_{i}^{\left(g+1\right)}=y_{i}^{\left(g\right)}-\varphi_{i}\left(\sigma^{\left(g\right)},{\text{y}}^{\left(g\right)}\right)$$

(160) $${\left(y_{i}^{\left(g+1\right)}\right)}^{2}={\left(y_{i}^{\left(g\right)}\right)}^{2}-\varphi_{i}^{\text{Il}}\left(\sigma^{\left(g\right)},{\text{y}}^{\left(g\right)}\right),$$

with constant normalized mutation strength σ* from Eq. (4) giving

(161) $$\sigma^{\left(g\right)}=\sigma^{*}\|{\text{y}}^{\left(g\right)}\|/N.$$

Two important issues need to be discussed. Firstly, the positional iterations are defined for a single component *i*. For large *N* however, it is not feasible to display each component individually. While the components will be iterated separately, the dynamics will be presented as a function of the residual distance *R* = ‖**y**^(*g*)^‖. Secondly, for the evaluation of $\varphi_{i}^{\text{II }}$ being a function of **y**^*(g)*^, the square root of the components ${\left(y_{i}^{\left(g\right)}\right)}^{2}$ has to be taken after iteration giving two solutions $\pm y_{i}^{\left(g\right)}$. As the corresponding terms of $\varphi_{i}^{\text{II }}$ and $D_{Q}^{2}(y)$ are even in $y_{i}^{\left(g\right)}$, both solutions are equivalent.

In the following, the deterministic iterations (159) and (160) using mutation strength rescaling (161) are compared to real optimization runs. For the initialization, **y**^(0)^ is chosen randomly such that ‖**y**^(0)^‖ = *R*^(0)^ for a given *R*^(0)^. The starting position is kept constant for consecutive runs of the same experiment. For the magnitude of *R*^(0)^ it is ensured that the strategy starts far enough away from the local minima landscape. Given Fig. 1 with *A* = 1, the farthermost local minimizer is at *y_i_* ≈ 3 with resulting $R\approx 3\sqrt{N}$ for *N*-components, such that $R^{\left(0\right)}=20\sqrt{N}>3\sqrt{N}$ is chosen.

**Fig. 8:**
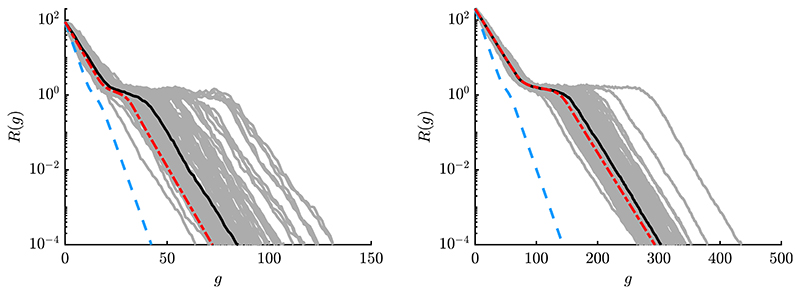
Comparison of real optimization runs with mean value dynamics using progress rates *φ_i_* via (157) [dashed blue] and $\varphi_{i}^{\text{II }}$ via (158) [dash-dotted red]. Gray lines show all 100 successful runs of Algorithm 1 and the black line shows the median thereof. The left plot shows (10/10, 40)-ES for *N* = 20 with $\sigma *=7({\hat{\sigma }}_{\text{sph}}^{*}=5.7)$ and the right one (100/100, 200)-ES for *N* = 100 with $\sigma^{*}=30\left({\hat{\sigma }}_{\text{sph}}^{*}=18.3\right)$. For both experiments *A* = 1, and *α* = 2*π* are chosen. The resulting success probability *P_S_* = 1.

Considering the choice of σ* one observes in experiments that larger mutation strengths (compared to a sphere-optimal σ*) increase the success probability *P_S_* of individual trials to converge to the global optimizer. This is due to the fact that large steps tend to overcome local attraction more easily. However, this comes at the expense of efficiency, since large steps are often overshooting the global optimizer. Therefore in Fig. 8, σ* is chosen larger than the sphere-optimal value ${\hat{\sigma }}_{\text{sph}}^{*},$ which can be obtained numerically from [5, Eq. (6.54)], but small enough to prevent negative progress. The aim was to obtain *P_S_* ≈ 1.

In order to aggregate the *R*^(*g*)^-data of multiple dynamic experiments, the median has shown to be a suitable measure of central tendency. The main issue is that due to fluctuations the *R*^(*g*)^-values of distinct ES-runs may differ by orders of magnitude, such that the mean yields biased results due to a skewed distribution. The median is more suitable in this case and a more stable measure.

In Fig. 8 one can observe three phases within the dynamics. First, linear convergence is observed for large *R*^(*g*)^-values, where the sphere function dominates. Then, a slow down is observed due to increasing effects of local attraction. For small *R*^(*g*)^-values, the ES descends into the global attractor basin and linear convergence can be observed again. One can see that the *φ_i_*-iteration (blue) shows by far too much progress compared to $\varphi_{i}^{\text{II }}$-iteration. This is due to the first order model, which does not include loss terms and overestimates the progress significantly, see also discussion of result (96). Iteration via $\varphi_{i}^{\text{II}}$ (red) shows good results compared to the median curve, especially for larger *μ* and *N* (right plot). Better agreement for large populations is also due to reduced fluctuation effects, which were neglected at the beginning of Sec. 5.

**Fig. 9:**
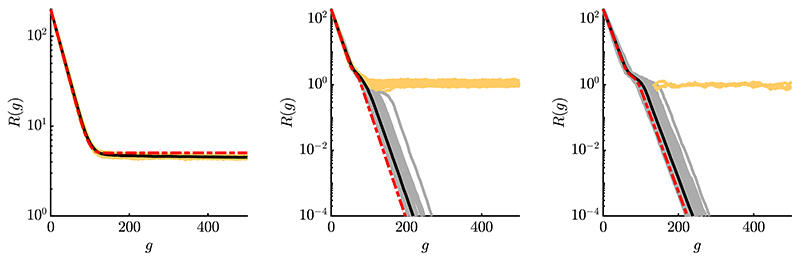
Variation of σ* for (100/100, 200)-ES for *N* = 100, *A* = 1, and *α* = 2*π*. From left to right σ* = {5, 18.3, 25}, with ${\hat{\sigma }}_{\text{sph}}^{*}=18.3$, and success rate *P_S_* = {0, 0.45, 0.97}. The experiment with σ* = 30 (*P_S_* = 1) was already shown in Fig. 8. Globally converging trials are shown in gray, and non-converging runs in light-orange. The median is taken over the globally converging runs, except for the left plot where none exist, in which the median over all unsuccessful runs is taken.

In Fig. 9 the effect of reduced σ* is investigated, which increases the probability of local convergence. The left plot shows σ* = 5 with no globally converging runs, as the mutation strength is too low. Technically, for constant σ* there is no local convergence as the algorithm never stops if *R* is not decreasing. Still, the experiments are stopped after some *g*-threshold is reached. The stagnating behavior of the ES around some *R^(g)^* can be illustrated using Fig. 3. For *σ* = 0.2 one has σ* = *σ N/R* ≈ 0.9, which is small compared to ${\hat{\sigma }}_{\text{sph}}^{*}\approx 5.7$. Both left and right progress components of Fig. 3 are significantly influenced by the local attraction region at *σ* = 0.2. While some components may be improved (positive value left), others are worsened (negative value right) resulting in a cumulative effect of *R*^(*g*)^-stagnation. One way out can be increasing *σ* (or equivalently σ*). However, the local minima landscape changes with changing *R* and arbitrary σ*-increase is not possible. Stagnation may appear at different σ* and *R*^(*g*)^-values depending on fitness and strategy parameters. For an active step-size adaptation, changing *σ* appropriately – without converging locally – poses a major challenge.

In the central plot of Fig. 9 roughly half of the runs are globally converging at increased $\sigma^{*}={\hat{\sigma }}_{\text{sph}}^{*}$. In this case the deterministic iteration follows a single converging path, as no fluctuations are modeled. The residual distance of the locally converging runs is reduced compared to ES-runs with σ* = 5. Note that the convergence speed is faster (steeper negative slope) for the globally converging runs compared to σ* = 30 of Fig. 8 due to sphere-optimal ${\hat{\sigma }}_{\text{sph}}^{*}$. However, this comes with the disadvantage of a lower *P_S_*, as more trials are converging locally. The right plot with σ* = 25 is similar to σ* = 30 of Fig. 8, but with several non-converging runs. Again, the ES convergence speed is faster, if σ* is chosen closer to ${\hat{\sigma }}_{\text{sph}}^{*}$, but shows a slightly reduced *P_S_* -value. The overall prediction quality of the iterative mapping (160) is good and the results affirm the expectation, that relatively large mutations are favorable to maximize *P_S_* on the Rastrigin function.

**Fig. 10:**
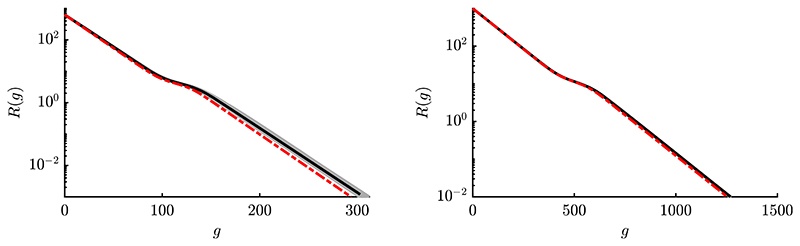
The left plot shows (1000/1000, 4000)-ES with σ* = 110 for *N* = 1000, *A* = 1, and *α* = 2*π*. The right plot shows (10000/10000, 20000)-ES with σ* = 400 for *N* = 10000 (same *α* and *A*), evaluated for 50 trials due to CPU resource restrictions.

To confirm the expectation that the approximation quality increases further for larger *μ* and *N*, experiments are shown in Fig. 10. First thing to notice is that positional fluctuations of the ES trials decrease further, such that nearly all runs show a similar *R*-dynamics. This is related to the intermediate recombination, see Eq. (34), as position **y**^(*g*+1)^ is obtained by averaging over a large number of individuals. One can see good agreement, but for the left plot there is still some room for improvement. This is related to truncation ratio *ϑ* = 1/4, such that the Taylor expansion point in Eq. (70) via function *g*(*x_i_*) is shifted by Φ^−1^(*ϑ*). For *ϑ* = 1/2 and even larger *N* and *μ* (right plot), very good agreement is observed.

## 6 Conclusion and outlook

In this paper the full first and second order progress rate analysis of the (*μ*/*μ_I_*, λ)-ES has been presented. In order to obtain closed-form expressions for *φ_i_* and $\varphi_{i}^{\text{II }}$ it was necessary to consider the large dimensionality and large population assumption. While the latter does not present a serious issue because large populations are needed to ensure global convergence, it was the key prerequisite to solve and simplify the expected value integrals. As the experiments have shown, the approximation quality of the progress rate expressions is rather good even for *N* as small as 20 and comparably small populations of *μ* = 10. For larger *N* and *μ* the approximation quality improves further, as expected. The first order progress rate result is able to model the local attraction effects on the Rastrigin function. This is a very important step, as all subsequent investigations in this paper are based on *φ_i_*-results. The second order progress rate derivation was needed to obtain additional loss terms completing the progress model, which was especially needed for larger mutation strengths and close to the global optimizer.

Using the progress rate expressions, the dynamics of the evolution process have been investigated. There is a good agreement between the iterations and real ES-runs using median aggregation of the residual distance *R* to the global optimizer. As has been shown, depending on the choice of the normalized mutation strength, one can model global as well as local convergence behavior. Additionally, one observes a trade-off between efficiency and success rate, as relatively large mutations have to be chosen to maximize the success probability.

The conducted experiments assume scale-invariance, i.e., the mutation strength is controlled by the residual distance *R*. This is in contrast to the full self-adaptive ES where *σ* evolves during the ES run either by mutative self-adaptation (SA), cumulative step-size adaptation (CSA), or Meta-ES. The incorporation of the self-adaptation process will be the next step completing the analysis of the (*μ*/*μ_I_*, λ)-ES on Rastrigin. To this end, the self-adaptation response (SAR) function must be derived. Combining *N* progress rates with the SAR function yields *N* + 1 evolution equations. In order to get manageable expressions that allow for analytic population sizing and expected runtime investigations, additional aggregation is needed. One possible approach would be the aggregation of individual parental *y_i_* components into the parental distance *R* modeling the expected progress as a function of the residual distance. This would reduce the number of evolution equations to two and making further analytic treatment more accessible. A first step in this direction has been done in [19].

Finally, the presented approach to model the ES-dynamics is based on mean value considerations. That is, fluctuations are not considered so far. Whether the approach presented can be extended to allow for the calculation of the global attractor convergence probability as a function of strategy and fitness parameters remains an open question.

## Data Availability

No data was used for the research described in the article.

## Footnotes

1 For independently distributed quality gain components *Q_i_*(*x_i_*) with finite mean and variance the Central Limit Theorem holds [10], if for some *δ* > 0 the Lyapunov condition

$$\underset{N\to \infty }{\lim}\frac{\sum_{i=1}^{N}\text{E}\left[{\left|Q_{i}-\text{E}\left[Q_{i}\right]\right|}^{2+\delta }\right]}{D_{Q}^{2+\delta }}=0$$

holds. The validation of the condition could be approached using Eqs. (24), (25), and (26) for the respective quantities by evaluating higher order moments.

2 Actually, using the result from (80) one could even calculate a closed-form second-order approximation for (69). However, the resulting formula would be rather complex.
